# Tunable Polymeric Scaffolds for Enzyme Immobilization

**DOI:** 10.3389/fbioe.2020.00830

**Published:** 2020-07-30

**Authors:** Andoni Rodriguez-Abetxuko, Daniel Sánchez-deAlcázar, Pablo Muñumer, Ana Beloqui

**Affiliations:** ^1^Nanomaterials Group, BRTA, CIC nanoGUNE, San Sebastián, Spain; ^2^PolyZymes group, POLYMAT and Department of Applied Chemistry (UPV/EHU), San Sebastián, Spain; ^3^Department of Applied Chemistry, University of the Basque Country, San Sebastián, Spain; ^4^IKERBASQUE, Bilbao, Spain

**Keywords:** enzyme-polymer hybrids, enzyme immobilization, polymeric supports, biocatalysis, nanocarriers, stabilization of enzymes

## Abstract

The number of methodologies for the immobilization of enzymes using polymeric supports is continuously growing due to the developments in the fields of biotechnology, polymer chemistry, and nanotechnology in the last years. Despite being excellent catalysts, enzymes are very sensitive molecules and can undergo denaturation beyond their natural environment. For overcoming this issue, polymer chemistry offers a wealth of opportunities for the successful combination of enzymes with versatile natural or synthetic polymers. The fabrication of functional, stable, and robust biocatalytic hybrid materials (nanoparticles, capsules, hydrogels, or films) has been proven advantageous for several applications such as biomedicine, organic synthesis, biosensing, and bioremediation. In this review, supported with recent examples of enzyme-protein hybrids, we provide an overview of the methods used to combine both macromolecules, as well as the future directions and the main challenges that are currently being tackled in this field.

## Introduction

The relevance of enzymes comprises numerous chemical processes in Nature, as they are the main actors in the metabolic machinery of each single organism. Moreover, enzymes are used in many industries and biotechnological applications due to their high efficiency, specificity, selectivity, and the possibility to carry out processes under the premises of Green Chemistry (Adrio and Demain, 2014). Unfortunately, the use of enzymes often presents several drawbacks, as they lose their functionality under those working conditions beyond their natural environment. Thus, enzymes can undergo denaturation throughout chemical degradation, physical unfolding, and aggregation caused by temperature or pH variations, organic solvents, or even the action of other enzymes (Balcão and Vila, 2015). In the last years, the formation of active and stable biocatalysts has been sought using assorted approaches, either through the alteration of the primary structure of the enzyme (i.e., rational design and directed evolution), by the immobilization of the enzyme on solid supports, through the chemical modification of the sequence of the protein, or by using combined approaches (Chapman and Stenzel, 2019). Molecular approaches, including computational modeling and structural biology, enable the modification of the active site or substrate/product channels within the enzyme, pursuing an enhancement of its bioactivity and stability (Beloqui and Cortajarena, 2020). Many of highly stable and genetically engineered enzymes are summarized in previous reviews (Arnold, 2018; Liu Q. et al., 2019). However, in this work, we tackle the use of polymeric scaffolds for the immobilization, protection, and stabilization of catalytic proteins.

Nillson and Griffin, in a pioneering work in 1916, were able to immobilize the invertase enzyme by physical adsorption to charcoal whilst maintaining its activity (Nelson and Griffin, 1916). Since then, aiming at overcoming the main drawbacks of the utilization of enzymes, i.e., low stability and costly production, many methodologies have been employed to tether enzymes to organic and inorganic materials. So far, poly(ethylene glycol) (PEG) is the most widely used and described polymer utilized to modify proteins. This polymer has been mainly placed to increase the solubility and/or stability of the hybrid system as a consequence of the shielding effects provided by the associated polymer (Cobo et al., 2015). Thus, PEG-protein hybrids show improved solubility, increased stability against degradation, increased circulation times, and prolonged biological activity (Krishna and Kiick, 2010). Fortunately, nowadays, the rapid growth of polymer chemistry offers a wealth of opportunities for the successful combination of enzymes with versatile natural or synthetic polymers. This combination gives rise to a huge diversity of structures and functionalities that embraces a wide range of applications in several research fields such as biocatalysis, biomedicine or biosensing. In the specific case of the field of biocatalysis, the benefits of anchoring synthetic polymers to catalytic proteins are multifold (Zhang Y. et al., 2015). Only through the combination of both (bio)materials, enzymes can reach unique regulated conformational properties such as nanostructured organization and supramolecular assembly. In this regard, the polymeric component can be just a mere solid architecture that provides suitable anchoring sites for the enzyme (widely used in the field of heterogeneous biocatalysis), or can participate actively in tailoring the properties of the enzyme in pursuit of a synergistic enhancement of the catalytic system.

**SCHEME 1:**
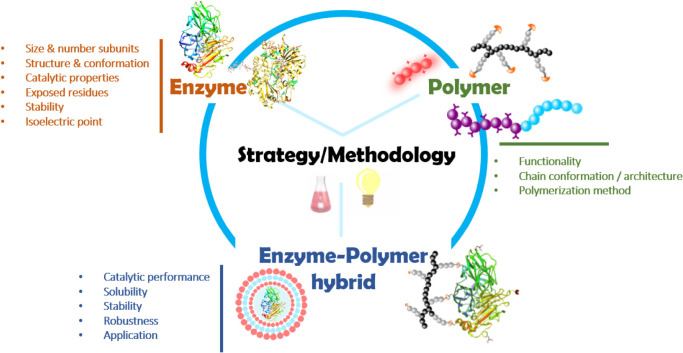
The synthesis of enzyme-polymer hybrids requires the selection of a rationally designed methodology in line with the selected polymeric material and the properties of the enzyme, which should keep the catalytic performance all along the synthesis procedure and in the eventual supramolecular structure. In this process, several parameters (some of which are highlighted in the scheme) need to be considered for a successful fabrication of enzyme-polymer hybrids.

The fabrication of enzyme-polymer hybrids is not a straightforward process, but a carefully designed strategy that should be optimized for each enzyme-polymer pair. Thus, in a well-designed three component system (enzyme, polymer, and methodology) (Scheme 1), not only the enzyme should retain its functionality, but also the polymeric material should provide the catalytic hybrid with the aimed features (e.g., recyclability, stability in organic solvents) to find potential synergistic properties. Hence, the whole procedure should consider several parameters beforehand. Obviously, the selection of the enzyme should be in line with the catalytic reaction that is pursued for the hybrid. In addition to the catalytic profile of the enzyme, other properties of the biomolecule should be also considered such as its size and number of monomers, its structure and conformation, the type and number of residues that are exposed to the environment, and its stability. As example, large proteins might not be suitable for their embedment into the porous network of the polymers. Moreover, it is of high importance to consider the isoelectric point of the enzyme that, besides being strongly related to the conformation and stability of the biomolecule, it can determine the feasibility of the conjugation reaction to the polymers, particularly when lysines are targeted.

Furthermore, the polymeric component also needs several considerations. The selection of the polymer and, in turn, of the methodology, will also rely upon the structure of the hybrid that is sought. Linear and water-soluble polymers are usually interesting for the stabilization of enzymes in solution (e.g., PEGlylated enzymes), whilst insoluble and more complex polymeric networks are used for the fabrication of hybrid heterogeneous biocatalysts (e.g., monoliths due to the high porosity or polymer films for biosensing due to the electrical conductivity features of some polymers) as is discussed below. In addition, the selection of the conjugation strategy that is carried out to couple the protein to the polymeric component must comply with the limitations set to retain the integrity of the biomolecules. In this regard, the addition of organic solvents at high concentration to the enzyme-polymer coupling reaction is usually inadvisable for the most of the enzymes. Further, the chain conformation of the polymer needs to be also evaluated, as large and bulky polymers might result in the hindering of the catalytic pocket of the enzyme, hence lowering its catalytic performance. All in all, the design of a successful experiment, in which a catalytically active, robust, and stable enzyme-polymer hybrid is fabricated, needs the careful study of multiple parameters.

On past decades, different enzyme-polymer immobilization methodologies have arisen, such as the adsorption or entrapment on/into solid polymeric particles, metal organic frameworks (MOFs), fibers, hydrogels, or monoliths; the encapsulation in polymersomes or polymeric capsules; and the preparation of cross-linked enzymes (Zdarta et al., 2018). Herein, we provide a short insight on the most used methods to combine both macromolecules, stressing the benefits/disadvantages of each approach. Moreover, this review attempts to cover the different enzyme-polymer designs and structures supported with recent examples from the literature. For the sake of clarity, we have classified by size the enzyme-polymer hybrids into four categories: single enzyme nanostructures; protein-polymer particles and capsules; micrometric hybrids; and millimeter structures.

## Strategies for the Fabrication of Enzyme-Polymer Hybrids

Over the past few years, numerous strategies have been developed for the fabrication of enzyme-polymer hybrids. The synthesis of the hybrid is facilitated either through the formation of covalent bonds or through non-bonding interactions between the enzyme and the polymer. Although it is not the main focus of this review, we provide a short description of the strategies used for the fabrication of the hybrids, those needed to ease the understanding of the formation of the enzyme-polymer hybrids described below. Thus, there are five main synthetic strategies that are herein exposed: covalent bonding, ionic and non-ionic interactions, physical entrapment, encapsulation, and affinity-based interactions. Whilst the first strategy means the formation of a strong bond between the two macromolecules, the driving force for the other four strategies is based on weak interactions such as Van der Waals interaction, hydrogen bonding, and ionic and affinity interactions. Importantly, it is worth mentioning that most of the hybrid structures that are herein detailed can be fabricated through more than one of the following strategies.

### Enzyme-Polymer Fabrication Through Covalent Bonding

**FIGURE 1:**
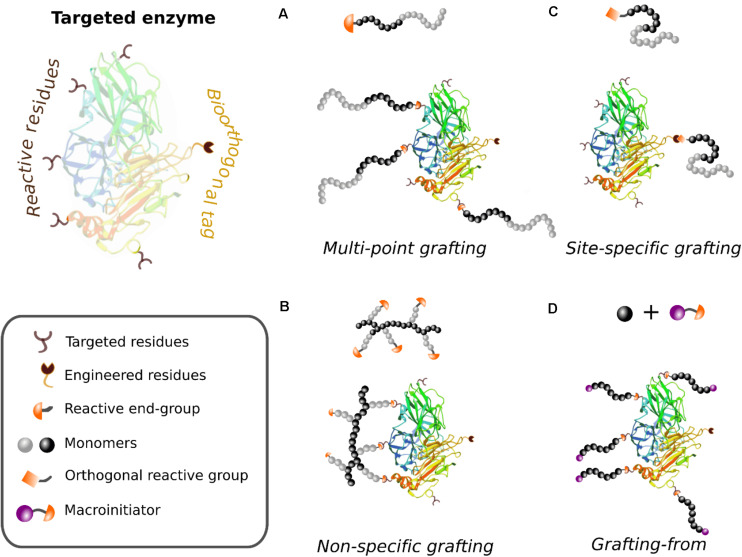
Schematic illustration of polymer-enzyme hybrids synthesized through different covalent-based methodologies. Strategies **(A–C)** require the synthesis of a preformed polymer (*grafting-to* approach) and **(D)** refers to the *in situ* synthesis of the enzyme-polymer hybrid (*grafting-from* approach). **(A)** Non-specific grafting of proteins, using polymers with a single reacting group and proteins with several target residues. **(B)** Multi-point strategy using polymers with multiple anchoring sites. **(C)** Site-specific grafting using a biorthogonal approach. **(D)** *Grafting-from* approach that entails the conjugation of the macroinitiator to the protein and the subsequent *in situ* polymerization after the addition of the monomers.

The covalent attachment of preformed polymers to a target enzyme is a widely used approach in the synthesis of enzyme-polymer conjugates (EPCs). Synthetic polymers can be designed with a large variety of architectures (e.g., linear or branched polymers) and functional end-groups to eventually react with several residues on the enzyme surface in a procedure that is generally known as *grafting-to* methodology (Figure 1) (Averick et al., 2015).

#### Non-specific Covalent Binding

Either single or multiple polymer chains can be tethered to the surface of the protein (Figure 1A). The number of anchored polymers relies on the nature and the number of amino acids that are targeted and on the steric issues that might be intrinsic to some polymer chains (e.g., bulky polymers or dendrimers), as is further discussed below. Alternatively, the use of branched polymers or networks allows the covalent modification of single enzymes from more than one unique point, increasing thereupon the stability of the enzyme (Figure 1B). This multi-point strategy is particularly relevant for the immobilization and stabilization of multi-subunit enzymes. Polymeric supports such as agarose, epoxy resins or polymethacrylate functionalized by glutaraldehyde (GA) or glyoxal groups are extensively used for the multipoint covalent immobilization of enzymes (Guisán, 1988).

Among all the amino acids of the protein, lysines and cysteines are likely the most targeted residues to carry out covalent bonding-based modifications of proteins. The predominance of lysine residues on the surface of the enzyme, usually exposed to the environment and thus accessible to the grafting polymer, facilitates the conjugation event. Different chemistries that lead to the formation of the amide bond can be employed, such as carboxylic acid–amine group reactions *via* carbodiimide chemistry (Hermanson, 2008) or amine–aldehyde addition–elimination reactions (Tao et al., 2004). Yet, the latter could trigger non-site-specific conjugations, modifying thereby other non-targeted residues, i.e., N-terminal amines, histidines, and tyrosines, in a minor degree (Turecek et al., 2016). In addition, the fact that all environmentally accessible lysines can react to some extent, leads to a poor control on the density of polymers and in their orientation on the surface of the proteins. Therefore, this approach generally results in a heterogeneous mixture of enzyme-polymer hybrids, provoking the decrease of the activity and the need of a laborious purification of the resultant mixture of the hybrids with different polymer loads (Canalle et al., 2010). On the other hand, free cysteines have raised as the most convenient target for the site-selective conjugation of native proteins. The highly nucleophilic sulfhydryl side chain group within cysteines can undergo alkylation with maleimides or iodoacetamides. In addition, they can be reacted with disulfide-containing reagents *via* exchange procedure (Jung and Kwon, 2016). Unfortunately, it is often challenging to target cysteine residues, as they are among the rarest residues, usually involved in disulfide bonds or buried in hydrophobic pockets. For this reason, other site-selective approaches that target less abundant residues are being developed.

#### Use of Biorthogonal Chemistry

For those experiments in which a high degree of control of the hybrid is a must, in terms of both the grafting density and the precise localization of the chains on the surface of the enzyme, highly efficient bioorthogonal chemistries are applied. Successful stories of site-selective bioconjugation have been achieved using thiol-ene (Jung and Kwon, 2016), alkyne-azide (Boyer et al., 2009), Diels-Alder (Sun et al., 2008) or Staudinger (Serwa et al., 2010) “click” reactions. The bioorthogonal reactions can be performed in presence of many nucleophiles, electrophiles, reductants, oxidants, or water without altering or affecting the evolution of the reaction, because they are not present in biological systems. The two biorthogonal moieties react selectively to each other under mild conditions. The formed covalent bonds are stable, and the byproducts innocuous. This strategy requires the insertion of exogenous functional groups (e.g., azide, hydrazine, alkyne, or allyl sulfide) to the protein. Such chemical handles can be introduced by the insertion of unnatural amino acids in the primary sequence of the protein through genetic engineering or by the direct labeling using other enzymes (e.g., subtiligase, microbial transglutaminase or farnesyltransferase) (Zhang Y. et al., 2018). On the other hand, the polymer component bears the complementary chemical handle in order to carry out a highly specific covalent reaction with the engineered enzyme under mild conditions (Figure 1C). Many hybrids such as single enzyme-polymer nanoconjugates (Wright et al., 2019), enzyme-MOF conjugates (Gkaniatsou et al., 2017), polymer brushes (Jiang and Xu, 2013), or polymer monoliths (Ma et al., 2019) have been successfully synthetized following this approach.

A large extent of proteins have been covalently bound by *grafting to* approach, mainly therapeutic proteins, but also enzymes with industrial interest and model proteins for proof-of-concept studies. In general terms, the main benefit of *grafting to* lies in that a broad spectrum of fully characterized preformed polymers can be attached to the enzymes, either site-specifically or randomly. However, there is a considerable issue with the attachment of bulky polymers or dendrimers that have intrinsic steric problems or hindered functional groups in their structure (Wang and Wu, 2018). Furthermore, a high grafting density is usually difficult to achieve using this approach (Carmali et al., 2017).

#### *Grafting-From* Approach

**FIGURE 2:**
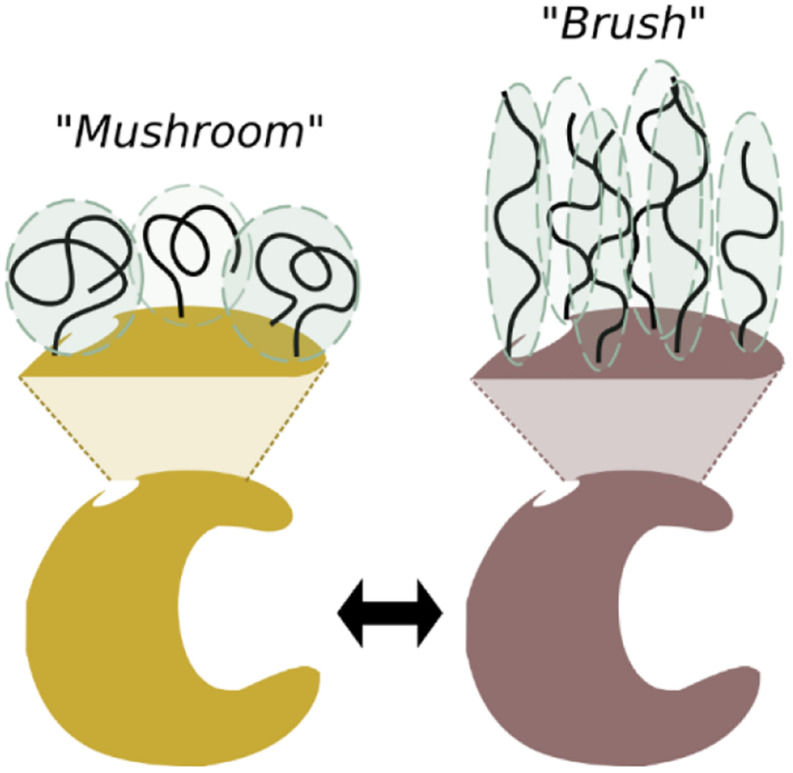
Polymers can be displayed over the enzyme surface in different configurations according to the grafting density. So called “mushroom” or “brush” configurations are achieved at low and high grafting density, respectively.

Aiming at increasing the grafting density and at targeting bulky polymers on the surface of the protein, a second approach known as *grafting from* arose (Messina et al., 2020). This approach consists in the *in situ* growth of the polymer, starting from the reaction initiators or chain transfer agents conjugated to the enzyme beforehand (Figure 1D). The *grafting from* approach demands mild polymerization conditions in order to, from one side, retain the catalytic properties of the enzymes and, from the other side, allow a precise control of the molecular weight of the polymer and the preservation of the chain-end functionality. In this regard, atom transfer radical polymerization (ATRP) and reversible addition fragmentation chain transfer polymerization (RAFT) are the most utilized techniques that fulfill the aforementioned requirements (Bontempo and Maynard, 2005; Boyer et al., 2007). Compared to *grafting to*, *grafting from* approach offers easier downstream purification as the monomers are much smaller than the conjugate itself. Moreover, at high monomer concentration, the entropic penalty of coupling two macromolecules is lowered, and more functional groups can be introduced. Therefore, the synthesis procedure can be tailored in order to tune the grafting density of the polymers on the surface and thus the configuration in which the polymers are presented. While low-density modifications lead to the “mushroom” configuration, the *grafting from* approach allows the highly dense configuration (“brush” configuration) (Figure 2), which usually leads to enhanced stability and higher solubility of enzymes in non-native environments (Ko and Maynard, 2018).

### Enzyme-Polymer Hybrid Fabrication Through Non-covalent Bonding

**FIGURE 3:**
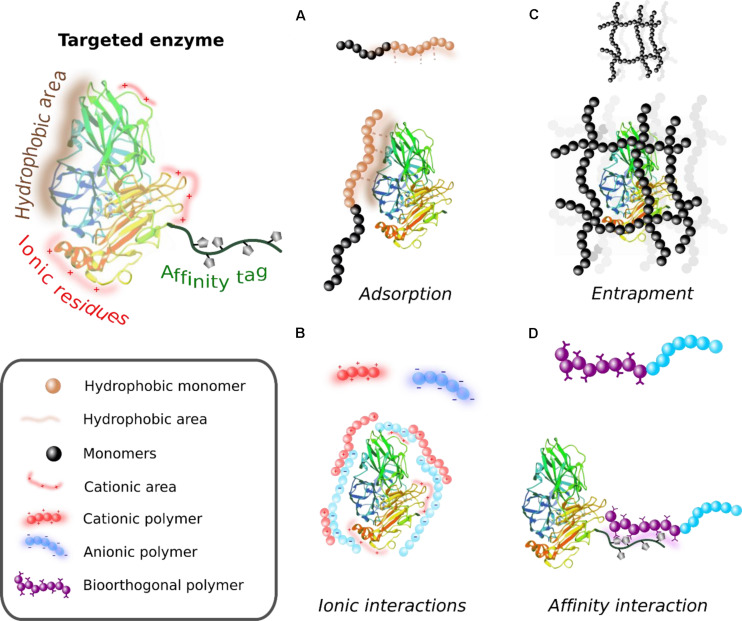
Schematic illustration of polymer-enzyme hybrids synthesized through different non-covalent based methodologies. **(A)** Adsorption of polymers on the surface of proteins. **(B)** Ionic interaction-based enzyme-polymer hybrids. **(C)** Entrapment of enzymes in the porous network of polymers. **(D)** Affinity-driven formation of enzyme-polymer hybrids.

Non-covalent enzyme-polymer conjugation strategies present an alternative route for enzyme modification. These approaches involve the adsorption of polymers on the surface of the enzyme through weak interactions, the entrapment of enzymes within a polymeric support, the encapsulation of enzymes into polymeric supramolecular assemblies, and the non-covalent specific bioaffinity binding of both components (Figure 3). As mentioned above, the selection of method, and hence the structure of the hybrid, must be in accordance with the application which the catalytic system will be involved in. Moreover, as discussed below, the physico-chemical properties (i.e., charge surface, polarity, molecular weight, and isoelectric point) and stability of the candidate enzyme is a key factor to be considered before the selection of the method.

#### Physical Adsorption

Physical adsorption is the most used non-covalent method due to its simplicity. The main interactions responsible for the adsorption process include Van der Waals forces, hydrogen bonds, and ionic and hydrophobic interactions (Figures 3A,B). These interactions are highly dependent on the environment, i.e., pH and ionic strength, and therefore can lead to protein leakage issues (Jesionowski et al., 2014). Thus, the effectiveness of the formation of the enzyme-polymer hybrid through adsorption entirely depends on the physico-chemical features of the enzyme and the support, such as charge, hydrophobicity, and the possibility to form hydrogen bonds. In this regard, the adsorption strategies may not offer the control over the spatial orientation of the proteins on the support, which can result in a decrease of the apparent biological activity of the system (Mateo et al., 2007). Notably, this approach is successfully used with lipases, well-known enzymes highly used in distinct industrial sectors. Most types of lipases have a peptide “lid” covering the active site. When the enzyme is adhered to hydrophobic interfaces, the “lid” changes to “open” conformation, enabling the access of the substrate to the active site and enhancing thereby the lipase activity dramatically, up to 50% of the activity of that shown by the native lipase (Palomo et al., 2002). There are many other examples of enzymes immobilized through adsorption on polymeric materials such as laccase (Labus et al., 2012), glucose oxidase (Koenig et al., 2016), carbonic anhydrase (Assarsson et al., 2014), and cellulase (Wang S. et al., 2013). Furthermore, there are interesting biopolymers for adsorbing enzymes, such as chitosan, calcium alginate, cellulose, agarose, or commercially available ion-exchange resins. Synthetic polymers with hydrophobic interfaces also form a large and diverse group of enzyme carriers. The most commonly used ones include poly(vinyl alcohol) (PVA), poly(*N*-methylolacrylamide) (PMAA), polypropylene (PP), polystyrene (PS), poly(hydroxybutyrate) (PHB) or poly(acrylonitrile) (PAN) (Jesionowski et al., 2014).

#### Physical Entrapment of Enzymes

The entrapment of enzymes within the cavities of polymeric matrixes, such as MOFs, electrospun fibers or hydrogels, is another noteworthy method for the fabrication of enzyme-polymer hybrids (Figure 3C). The enzyme is trapped either during (*in situ* approach) or after the assembly of the polymers into networks or supramolecular structures. The entrapped enzymes typically show enhanced stability compared to those located on the surface through adsorption (Hiep Nguyen and Kim, 2017). The microenvironment, i.e., pH, polarity or amphiphilicity, and the matrix pore size can be adjusted although inefficient mass transfer is generally observed for the enzymes deeper entrapped (Sassolas et al., 2012). In contrast, large pore sizes of the matrix support are likely to suffer from enzyme leakage. There are several assembled supramolecular polymeric structures utilized for the embedment of enzymes [i.e., polymersomes, reverse micelles, polyion complex vesicles (PICsomes), and layer-by-layer capsules]. The most commonly utilized procedures are based on copolymer self-assembly, layer-by-layer (LbL) assembly, and emulsion polymerization synthesis (Cuomo et al., 2019). Additionally, polymerization-induced self-assembly (PISA) synthesis approach for enzyme encapsulation is standing out from the rest of methods because of its simplicity, mild assembly conditions, and broad versatility. Furthermore, the self-assembly of amphiphilic block copolymers in presence of enzymes is carried out in water. In this way, the hydrophobic interactions between the nonpolar blocks of the growing polymers are enhanced, giving rise to the spontaneous assembly into ordered structures such as polymersomes or reverse micelles, leaving the enzymes confined inside (Matoori and Leroux, 2020). Moreover, this assembly strategy can be combined with different techniques of polymerization, i.e., RAFT, ROMP, ATRP, anionic polymerization, and ring opening polymerization (Varlas et al., 2019).

#### Affinity-Based Approach

Affinity-based methods are also utilized for the controlled and site-specific modification of enzymes on/in polymeric materials using specific non-covalent interactions (Figure 3D). Several are the benefits of using affinity immobilization methods. The affinity-based interactions are usually reversible, which enables the recycling of the supporting material when the attached enzyme loses activity. Also, despite being a reversible bonding, the interactions between the enzyme and the polymeric material are specific and stable under usual working conditions. Furthermore, natural or artificial epitopes or tags are strategically inserted in the protein in order to promote a favorable orientation, which usually enhances the catalytic performance of the enzyme. Several examples of peptide-tags that are artificially introduced in recombinant proteins can be found in the literature [e.g., cellulose-binding domains (CBDs) (Dai et al., 2017), SNAP Tag (Fang et al., 2019), matter-tag (Dedisch et al., 2020), solid-binding peptides (Care et al., 2017), or FLAG tag (Vishwanath et al., 1997)]. The most used polymers bear affinity tags such as nitrilotriacetic acid (NTA) functionalization or avidin/streptavidin motifs, which are used to tether His-tagged or biotinylated proteins, respectively. Indeed, enzyme immobilization on polymers mediated by chelated transition metals (Coulet et al., 1981) has been proven as one of the most convenient methodologies for enzyme immobilization and purification (Yakup Arica and Bayramoglu, 2004). Metal-ligand coordination guided immobilization lowers the mass transfer resistance of the substrate/product. Moreover, the biocatalyst can be easily recovered (Bayramoglu et al., 2010). The variety of affinity-based approaches is huge and is continuously growing [e.g., cellulose binding domains-cellulose and chitin (Kowsari et al., 2014), glycosylated polymers-lectin (López-Gallego et al., 2012), calmodulin protein domain-phenothiazine ligands (Daunert et al., 2007), etc]. For further details, we recommend Barbosa et al. (2015), where the main domains for affinity enzyme immobilization are reviewed.

## Enzyme-Polymer Hybrids

Enzyme-polymer hybrids can be assembled into a plethora of structures, from the simplest enzyme-polymer linear structures to the complex supramolecular polymersomes, ranging from the nanoscale to the macroscopic size. As mentioned before, the selection of the appropriate polymers, guided by the convenient methodology, will address the morphology and the size of the achieved catalytic system. From the catalysis perspective, smallest hybrids, with less non-catalytic material, can achieve much higher enzyme loading capacity and significantly lower diffusion issues. However, larger structures usually stabilize the enzyme in a major degree. In this review, we summarize the most relevant structures found in the literature, sorted out by the size of the enzyme-polymer hybrid, from nanobiocatalysts to micro- and macrosystems.

### Assembly Into Nanobiocatalysts (10–200 nm)

Recently, joint efforts in the fields of biotechnology, polymer chemistry, and nanotechnology have led to significant progresses in the synthesis and characterization of advanced nanobiocatalysts. Hence, protocols have been stablished for the fabrication of efficient single enzyme nanostructures, such as enzyme-polymer conjugates (EPCs) or single-enzyme nanogels (SENs), repeatedly used in the field of therapeutics and biomedicine (examples collected in Table 1). Nanobiocatalysts can be prepared through the attachment of enzymes *via* classical methods, i.e., covalent conjugation or entrapment, giving rise to hybrid units that comprise one single enzyme, or in combination with nanometric supports such as polymeric nanoparticles and capsules that can be loaded with several biomacromolecules. The main benefits from nanobiocatalysts lie in the high enzyme loadings that are achieved per weight of non-catalytic material. This fact is usually translated into a high catalytic performance of the system. Yet, recycling issues are inherent to the use of nanometric systems.

**TABLE 1:** Summary of the single enzyme nanostructures tackled in this work (from 5 to 40 nm).

Structure	Non-enzymatic moiety	Enzyme	Immobilization method	Hybrid size (nm)	Characteristics (regarding activity)	Application	References
EPCs^1^	PCBMA^3^	α-CT^12^	Covalent binding	NA*	Enhanced affinity/thermostability	Potential protein therapeutics	Keefe and Jiang, 2012
	PCBMA, POEGMA^4^, PDMAEMA^5^, PQA^6^, PSMA^7^	α-CT		5–30	Broaden functional pH	Emulating chaperones for modulating enzyme folding	Baker et al., 2018
	PNAM^8^, pOEGMA	Lyz^13^		4.7–6.4	Enhanced activity/solubility	Possible life science applications	Morgenstern et al., 2018
	PSMA	α-CT, UOX^14^, AChE^15^, Lyz		13 (α-CT)	Enhanced activity/solubility	Model for preserving the surface charge	Baker et al., 2019
	PDMAPA^9^	TL^16^		NA*	150% vs. free enzyme	Polymer effect on activity	Kovaliov et al., 2018
SENs^2^	PAA^10^	HRP^17^	*In situ* encapsulation	9–11	Thermostable at 65°C	Model for thermostability	Yan et al., 2006
	PAA	CALB^18^		13–40	Stable at 60°C (DMSO^27^)	Stability in organic solvents	Ge et al., 2008, 2009
	PAA	HRP, GOx^19^		10–20	No activity loss	Stamped surfaces	Beloqui et al., 2016
	PAA	HRP, SOD^20^, CP-3^21^		15, 12, 20	Activity *in vitro*	Enzyme delivery	Yan et al., 2010
	NH2-PAA	CP-3		13	Low	Enzyme delivery	Gu et al., 2009
	PAA	GOx, HRP, PfE^22^, β-Glu^23^, CALB, TvL^24^,CAT, AOx^25^,		10–20	50–100%	Model for stability	Beloqui et al., 2018
	NH2-Imidazole-PAA	HRP		10	No activity loss	Heterogeneous catalysts	Rodriguez-Abetxuko et al., 2018
	Imidazole, PEG	Cas9^26^		25	Not comparable	Gene editing	Chen et al., 2019
	PCB^11^	HRP		<40	3-fold (*k*_cat_)^28^ vs. free	Bioremediation	Zheng et al., 2019
	PCBMA	Uricase		*ca.* 30	100% (2 h at 65°C)	Mitigating immune response	Zhang P. et al., 2015

*^∗^Not available; ^1^enzyme polymer conjugates; ^2^single enzyme nanogels; ^3^poly(carboxybetaine methacrylate); ^4^poly[oligo(ethylene glycol) methyl ether methacrylate]; ^5^poly(dimethylamino)ethyl methacrylate; ^6^poly(quaternary ammonium methacrylate); ^7^poly(styrene maleic anhydride); ^8^poly(N-acryloylmorpholine); ^9^N-[3-(dimethylamino)propyl]acrylamide (DMAPA); ^ 10^polyacrylic acid; ^11^polycarboxybetaine; ^12^α-chymotrypsin; ^13^lysozyme; ^14^urate oxidase; ^15^acetylcholinesterase; ^16^thermomyces lanuginose lipase; ^17^horseradish peroxidase; ^18^Candida antarctica lipase B; ^19^glucose oxidase: ^20^superoxide dismutase; ^21^caspase; ^22^Pseudomonas fluorescens esterase; ^23^β-glucosidase; ^24^Trametes versicolor laccase; ^25^alcohol oxidase; ^26^CRISPR associated protein 9; ^27^dimethyl sulfoxide; ^28^catalytic constant.*

#### Single Enzyme Nanostructures

##### Enzyme-polymer conjugates (EPC) (<40 nm)

In last years, the covalent conjugation of synthetic polymers, either through *grafting to* or *grafting from* approach, to enzyme surfaces has become a common and very successful way for the generation of active and stable single enzyme polymer conjugates (Figure 1A). Lots of synthetic polymers [e.g., poly(ethylene glycol) (PEG), poly(*N*-isopropylacrylamide) (PNIPAAM), poly(carboxybetaine methacrylate) (PCBMA), poly(oligo(ethylene glycol) methyl ether methacrylate) (POEGMA), poly(quaternary ammonium methacrylate) (PQA), poly(styrene maleic anhydride) (PSMA), to cite some], and biopolymers (e.g., trehalose-based glycomers or elastin-like polypeptides), have been utilized for equipping many enzymes with novel functionalities and hence with broad applicability (Wright et al., 2019). Gauthier and Klok (2010) reviewed most of the enzyme-protein conjugates present in literature until 2010, concluding that the polymer conjugation often diminishes to some point the initial bioactivity, although the stability is greatly improved. However, since 2010, this field has been remarkably evolved in scope and complexity. We currently know that there are several parameters related to the nature of the polymer, i.e., charge, hydrophobicity, length, and functionality, which have a clear impact over the performance of the catalytic hybrid system and need to be precisely controlled. Indeed, several works evaluating the effect of these parameters on the bioactivity have been published in the last years (Table 1). Thus, Baker et al. (2018) synthetized different chymotrypsin (α-CT)-polymer conjugates varying polymer charge, hydrophobicity, and molecular weight. They conjugated zwitterionic PCBMA, neutral POEGMA, neutral to positive poly(dimethylamino)ethyl methacrylate (PDMAEMA), positive quaternary ammonium ion-containing polymers (PQA), and negative poly(styrene–maleic anhydride) (PSMA) of three different chain lengths each. A *grafting from* approach was followed, targeting the lysine residues of the α-CT enzyme. After a careful study and characterization, they observed that the charge of the polymer had a strong influence in the activity. The conjugation of positively charged polymers (PDMAEMA and PQA) to the enzyme surface increased the catalytic efficiency in acidic environments, which was translated into an increase of the affinity (ca. 25%) toward the negatively charged substrate. This effect is attributed to long-range electrostatic interactions between the polymer and the histidine located in the active site, which fosters the catalytic efficiency. These results point out the importance of designing a suitable environment for each enzyme using tailored polymers that need to be designed for each particular case (Baker et al., 2019).

The effect of the hydrophobicity of the polymeric chain on the activity and stability of the hybrid has also been studied. The synthesis and characterization of α-CT-PCBMA (poly(carboxybetaine methacrylate)) enzyme-polymer conjugates (EPCs) elucidated interesting remarks (Keefe and Jiang, 2012). Apparently, the high hydrophilicity of PCBMA strengthens the hydrophobic interactions that hold the tertiary structure of the protein. Moreover, the affinity of the enzyme toward the peptide-based substrate was increased by a 30% due to stronger enzyme-substrate hydrophobic-hydrophobic interactions. Moreover, the effect of the PCBMA polymer in the biocatalyst goes beyond the enhancement of the catalytic performance. They also observed that the α-CT-PCBMA hybrid was really stable compared to the native enzyme at high temperatures (almost 100% of the activity conserved at 50°C) and in the presence of denaturing agents.

Furthermore, besides the charge and hydrophobicity of the polymers, the position of the conjugation site, the grafting density, and the molecular weight of the attached polymer are other parameters that have a clear impact on the activity and stability of EPCs. Obviously, the tethering of long and bulky polymers close to the substrate tunnel or catalytic cavity of the enzyme is undesirable in order to avoid the obstruction of the substrate diffusion to the catalytic site. A similar effect occurs with heavily dense grafted hybrids. In this regard, Morgenstern et al. (2018) evaluated the influence of the polymer molar mass and protein conjugation degree on the solubility, aggregation behavior, and *in vitro* activity of poly(*N*-acryloylmorpholine) (PNAM)- and POEGMA-based lysozyme conjugates synthetized by *grafting-to* approach. The conjugates with larger polymer chains, as well as the polyvalent conjugates, showed a reduced catalytic activity, attributed to the shielding of the catalytic site by the polymer. However, the monovalent conjugates with the shortest polymeric chains showed moderately increased activities compared to native lysozyme (Morgenstern et al., 2018). However, it has been demonstrated that a well-controlled grafting density and polymer length, i.e., using *grafting-from* approach, can trigger the enhancement of the catalytic activity. Thus, the controlled grafting of positively charged polymers close to the active center can increase the effective concentration of the negatively charged substrates and thereby boost the activity of the biocatalyst (Kovaliov et al., 2018). These examples showcases that the synthesis of successful EPCs requires a careful design of the parameters of the polymer but also a suitable synthesis strategy that allows the achievement of new opportunities to the biocatalyst.

##### Single enzyme nanogels (SENs) (<40 nm)

Single enzyme nanogels have arisen as a promising technology in which enzymes are trapped individually by a thin hydrogel layer. The synthesis is performed *in situ*, giving rise to core-shell-like structures, in which the biomolecules remain in the core and the polymer works as a protective shell. These structures generally show catalytic performances close to that exhibited by the native enzyme. Also, a clear enhancement of the stability at high temperatures and in presence of organic solvents is observed. The synthesis consists of a two-step procedure. First, vinyl groups are anchored to the protein through the covalent modification of lysine residues. Thereafter, the *in situ* free radical polymerization is performed on the surface of the protein by the co-addition of monomers, crosslinkers, and the acryloylated enzyme at room temperature (Yan et al., 2006). The mechanism that undergoes nanocapsule formation was elucidated by molecular simulation. It was demonstrated that the monomers are locally concentrated around the enzyme surface prior to polymerization, *via* hydrogen bonding, additional static electronic forces, and hydrophobic interactions (Ge et al., 2008). The thermostability of several enzymes was significantly enhanced using this strategy. As example, horseradish peroxidase (HRP) nanogels could conserve the 80% of its initial activity after 90 min of incubation at 65°C, while native HRP was totally inactivated under the same conditions. Moreover, in presence of organic solvents, the activity of HPR was also retained due to the hydrophilic environment formed by the nanogel. The authors attributed the enhanced thermal stability of the HRP nanogel to the multiple interactions between the polymeric network and the protein (Ge et al., 2009; Beloqui et al., 2016).

**FIGURE 4:**
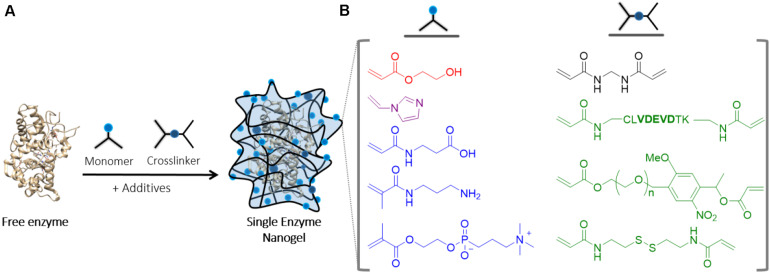
Synthesis of single enzyme nanogels. **(A)** Fabrication procedure of single enzyme nanogels by *in situ* radical polymerization. **(B)** We showcase some of the most used monomers and crosslinkers. Selected monomers can provide hydrophilicity (red), the ability to complex metal cations (purple) or tune the ionic interactions within the polymeric shell (blue). Moreover, the use of labile crosslinkers allows the release of the protein under external stimuli (presence of a peptidase, light or redox reagents for the examples drawn in green).

The synthesis procedure of SENs has been optimized in order to broaden the applicability of the technology to a wide range of enzymes and monomers (Figure 4A). It has been demonstrated that the addition of small amounts of sucrose triggers the reduction of hydrophobic/hydrophilic repulsion forces between monomers and enzymes, increasing thereby the concentration of the monomers on the surface of the protein and thus enhancing the encapsulation yield to almost 80% (Beloqui et al., 2018). It has been also observed that it is possible to control the thickness of the capsule, regardless the sort of protein utilized, only varying the monomer concentration. Importantly, it was found that layers with more than 2 nm thickness are detrimental for the enzymatic activity. Thicker layers lead to mass transfer issues. In more recent examples, new functionalities have been introduced into the polymeric shell, i.e., hydroxyl, amino, carboxyl, and imidazole groups (Figure 4B), which have been further utilized as building blocks for the synthesis of heterogeneous catalysts for biocatalysis or biosensing (Rodriguez-Abetxuko et al., 2018, 2019a,b). Indeed, SENs, which were initially conceived to enhance the stability of the enzyme, have been also applied in different fields. Zwitterionic capsules, which confer extreme hydrophilicity, antifouling properties, and non-immunogenicity, have been utilized for therapeutics and the removal of pollutants (Zhang P. et al., 2015; Zheng et al., 2019). Moreover, the small size of SENs makes them an interesting vehicle for protein delivery *in vivo* (Gu et al., 2009; Yan et al., 2010; Biswas et al., 2011). In this regard, Cas9 ribonucleoprotein complex was successfully encapsulated and delivered for gene-editing experiments using degradable nanocapsules (Chen et al., 2019). Certainly, the advances achieved in the lasts years derive into the encapsulation of enzymes into complex and smart polymers that might enable the inclusion of this technology into other hot fields, such as the chemoenzymatic catalysis or biohybrid light emitting diodes (Rodriguez-Abetxuko et al., 2020).

#### Enzyme Immobilization Onto/Into Polymeric Nanomaterials

In the last decades, polymeric nanomaterials with distinct morphologies and properties have been widely used as carrier platforms to confine several enzymes in small volumes. Enzymes can be either tethered to the surface of the polymers, encapsulated into hollow structures, or embedded into the network of porous polymers. This strategy results interesting for multitude of biotechnological applications such as biocatalysis, bioseparations, imaging, biosensing, *in vitro* biotransformation, drug delivery or therapy (Hola et al., 2015; Bosio et al., 2016) (examples collected in Tables 2, 3). In this section, we have revised the methodologies and structures used for the confinement of enzymes in different nanomaterials up to date. We have classified the hybrids attending to the relative arrangement of the enzyme and the polymeric material: enzymes bound to the surface of the polymer through (non)covalent interactions; enzymes encapsulated in the core of polymeric supramolecular structures; and enzymes entrapped into a specific type of nanoscale polymeric networks, namely, nanoMOFs.

**TABLE 2:** Summary of enzyme-polymer nanohybrids in which the enzyme is displayed to the environment.

Structure	Non-enzymatic moiety	Enzyme	Immobilization method	Hybrid size (nm)	Characteristics (regarding activity)	Application	References
Micelle corona	Pluronic F127	OPH^8^	Covalent (NHS^24^-Lys)	120	1.5-fold increase vs. free enzyme (*k*_cat_^28^)	Bioremediation	Suthiwangcharoen and Nagarajan, 2014
	POEGMA^2^	HRP^9^, Est^10^	Affinity Ni^2+^/His tag	130–220	3-fold decrease vs. free HRP (*k*_cat_)	Proof of concept study	Keller et al., 2017
Dendrimer	PBA^3^-modified PAMAM^4^	Cas9^11^/sgRNA^12^, trypsin, β-Gal^13^, Lyz^14^, Cyt c^15^, HRP, and RNase A	Nitrogen-boronate complexation Cation-π interactions Ionic interaction	100–200	>80% maintained after delivery (β-Gal)	Cytosolic enzyme delivery and gene editing	Liu C. et al., 2019
	PAMAM + dipicolylamine/zinc (II)	β-Gal, RNase A, SOD^16^, α-CT^17^, Lyz, HRP, trypsin	Ionic and coordination interactions (DPA^25^/Zn^2+^ and imidazole/amino)	100–200	Nearly 90% (HRP)	Cytosolic enzyme delivery	Ren et al., 2020
NP	PdPt bimetallic core and PDA^5^ shell	CALB^18^/CALA^19^ /OPH	Bioadhesion-inspired strategy	*ca.* 50	99% yield and 98% ee^29^ (CALB). 78% yield and 93% ee (CALA).	DKR and degradation of OP nerve agent	Gao et al., 2020
	Iron oxide, PGMA^6^	ADH^20^, TL^21^	Covalent (GA^26^)	*ca.* 70–160	97% yield in 60 min > 99% ee (R). 14 times recycled 80% activity. Thermostable at 70°C for 12 h.	Model for heterogeneous catalysis	Ngo et al., 2012
	Iron oxide, PGMA	OASS^22^	Affinity (Ni-NTA^27^)	85	55% activity in the 10th cycle.	Unnatural aa synthesis	Vahidi et al., 2016
Self-assembled EPC^1^	PHPMA^7^ core	GOx^23^/HRP	Covalent, adsorption	60–68	Fivefold enhanced cascade activity	Glucose detection	Chiang et al., 2020

*^1^Enzyme polymer conjugate; ^2^poly(oligo(ethylene glycol) methyl ether methacrylate); ^3^phenylboronic acid; ^4^poly(amidoamine); ^5^polydopamine; ^6^poly(glycidyl methacrylate); ^7^poly(N-(2-hydroxypropyl) methacrylate)); ^8^organophosphate hydrolase; ^9^horseradish peroxidase; ^10^esterase; ^11^CRISPR associated protein 9; ^12^single guide RNA; ^13^β-galactosidase; ^14^lysozyme; ^15^cytochrome c; ^16^superoxide dismutase; ^17^α-chymotrypsin; ^18^Candida antarctica lipase B; ^19^Candida antarctica lipase A; ^20^alcohol dehydrogenase; ^21^thermomyces lanuginose lipase; ^22^O-acetylserine sulfhydrylase; ^23^glucose oxidase; ^24^N-hydroxysuccinimide; ^25^dipicolylamine; ^26^glutaraldehyde; ^27^nitrilotriacetic acid; ^28^Catalytic rate constant; ^29^enantiomeric excess.*

**TABLE 3:** Examples of enzyme-polymer supramolecular nanohybrids in which the enzyme is confined in the inner cavity.

Structure	Non-enzymatic moiety	Enzyme	Immobilization method	Hybrid size (nm)	Characteristics (regarding activity)	Application	References
Reverse micelle	BHDC^3^	α-CT^20^	Encapsulation	11	Enhanced efficiency in water/BHDC^37^/benzene	Biotransformation	Moyano et al., 2010
	CTAB^4^/AuNP	Lipase	Encapsulation	*ca.* 40	3.5-fold increase vs. no NP RMs^38^	Model for nanoreactor	Maiti et al., 2011
	PS-based^5^	plE^21^, bCA^22^, Lyz^23^	Encapsulation/Affinity binding/adsorption	37–50	Activity maintained in the transport	Selective transport	Gao et al., 2018
Polymersome	PMOXA^6^-PDMS^7^-PMOXA	AGE^24^/NAL^25^ /CSS^26^	NAL, CSS adsorption. AGE encapsulated	110	3.9-fold compared to polymersome without membrane channel	Model for reaction segregation	Klermund et al., 2017
	PS-*b*-PAA^8^	Trypsin	Encapsulated	30–250	2 orders of magnitude increase in enzyme efficiency vs. bulk	Model for confinement	Chen et al., 2009
	PEG^9^-*b*-PS PEG-*b*-PSBA^10^	CALB^27^	Encapsulated	200–400	Increased with permeability	Model for nanoreactors controlled permeability	Kim et al., 2009
PICsome^1^	PEG-*b*-P(Asp)^11^, P(Asp-AP)^12^	L-ASNase^28^	Encapsulation	100	Prolonged activity *in vivo*	Model for therapy	Sueyoshi et al., 2017
	PEG-P(Asp), Homo-P(Asp-AP)^13^	β-Gal^29^	Encapsulation	*ca.* 100	2-fold K_m_^app39^ vs. free enzyme	Enzyme delivery	Anraku et al., 2016
MOFs^2^ NPs	ZIF-8^14^	CAT^30^/GOx^31^	Covalent bond on ZIF surface by GA^36^	*ca.* 200	Activity preservation	Photodynamic therapy	You et al., 2019
	CC-ZIF^15^ coated with cancer cells membrane	Cas9^32^	*In situ* encapsulation	120	Activity preservation, fast release of Cas 9	CRISPR^40^/Cas 9 delivery	Alyami et al., 2020
	ZIF-8	φ29 DNA polymerase	*In situ* encapsulation	*ca.* 90	Enzyme shielding, activity preserved	Rolling circle amplification (RCA) *in vivo*	Zhang et al., 2019a
	ZIF-8 and UiO-66^16^	Taq polymerase	Infiltration into MOFs	*ca.* 1000	Increase of sensitivity and efficiency	Polymerase chain reaction (PCR)	Sun et al., 2020
	ZIF-8	Lipases	*In situ* encapsulation	NA*	High activity (15 days storage)	Model for biocatalysis	Pitzalis et al., 2018
	ZIF-8	Cyt c^33^, HRP^34^, CALB	*In situ* encapsulation	20–30	Activity preserved even in organic solvents	Enhancing stability	Wu et al., 2017
	ZIF-8	Cyt c	Infiltration into MOFs	*ca.* 1000	*K*_m_^app^ reduced by ∼50%, 1.4-fold increased sensitivity	Electrochemical biosensor of H_2_O_2_	Zhang et al., 2017
	ZIF-8 coating of PVP^17^	β-Gal, CP-3^35^	Encapsulation	70–200	Activity preserved	Biomedical application	Chen et al., 2018
COF/MOF NPs	COF42-B(BBTH/TB)^18^ ZPF-2^19^ or ZIF-90	GOx and CAT/GOx	*In situ* encapsulation	*ca.* 1.5	Increase activity	Model for cascade reaction	Li et al., 2020

*^∗^Not available; ^1^polyion complex vesicles; ^2^metal organic frameworks; ^3^benzyl-N-hexadecyldimethylammonium chloride; ^4^cetyltrimethyl-ammoniumbromide; ^5^polystyrene; ^6^poly(2-methyl-2-oxazoline); ^7^polydimethylsiloxane; ^8^polyacrylic acid; ^9^polyethylene glycol; ^10^poly(ethylene glycol)-b-poly(styrene boronic acid); ^11^PEG- poly(α,β-aspartic acid); ^12^poly([5- aminopentyl]-α,β-asoartamide); ^13^homo-([5-aminopentyl]-α,β-aspartamide); ^14^zeolitic imidazolate frameworks; ^15^CRISPR/Cas9-ZIF; ^16^Universitetet i Oslo-66; ^17^polyvinylpyrrolidone; ^18^covalent organic frameworks-42-B (2, 5- Bis(butenyloxy)terephthalohydrazide/1,3,5-triformylbenzene); ^19^zeolitic pyrimidine framework; ^20^α-Chymotrypsin; ^21^porcine liver esterase; ^22^bovine carbon anhydrase; ^23^lysozyme; ^24^N-acyl-D-glucosamine 2-epimerase; ^25^N-acetylneuraminate lyase; ^ 26^CMP-sialic acid synthetase; ^27^Candida antarctica lipase B; ^28^L-asparaginase; ^29^β-galactosidase; ^30^catalase; ^31^glucose oxidase; ^32^CRISPR associated protein 9; ^33^cytochrome c; ^34^horseradish peroxidase; ^35^caspase 3; ^36^glutaraldehyde; ^37^benzyl-n- hexadecyldimethylammonium chloride; ^38^reverse micelles; ^39^apparent Michaelis constant; ^40^clustered regularly interspaced short palindromic repeats.*

##### Immobilization on the surface of the nanomaterial

**FIGURE 5:**
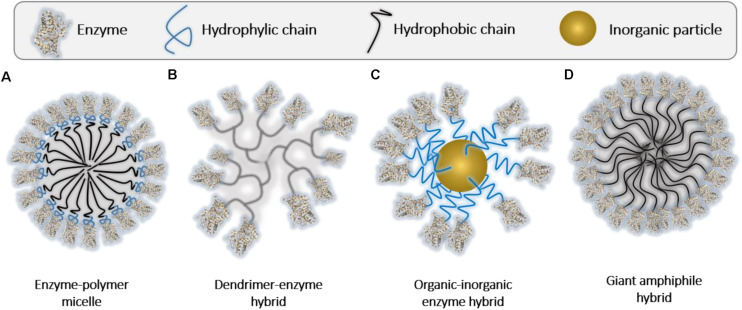
Assembled enzyme-polymer supramolecular hybrids in which the enzyme is displayed to the environment. **(A)** Enzyme-polymer micelles. **(B)** Dendrimer-enzyme hybrids. **(C)** Organic-inorganic enzyme hybrids. **(D)** Giant amphiphile structures.

Polymeric nanostructures, i.e., micelles, dendrimers, and metal/polymer nanoparticles, have a large specific surface area, which makes them ideal to accommodate a dense layer of enzymes that will remain exposed to the environment (Figure 5). While this is an easy and convenient approach to concentrate the biocatalysts in small volumes, the protection provided by the polymer component is in this case very limited compared to the hybrids described in the next sections. In this review, due to their high significance and number of examples in the literature, we want to highlight the hybrids fabricated using four sorts of polymeric nanoarchitectures, namely, polymeric micelles, dendrimers, polymeric nanoparticles, and so called giant amphiphile hybrids.

##### Polymeric micelles

Polymeric micelles (*ca*. 100–200 nm) are nanoscopic core-shell structures formed by amphiphilic block co-polymers in which the hydrophobic part is located in the inner core and hydrophilic part outward. Hence, enzymes can be conjugated to the outer hydrophilic part of preformed micelles, modifying the micelle corona (Figure 5A). The micelles usually generate an appropriate environment for the enzymes, enhancing their physicochemical and biological properties. As example, Keller et al. (2017) successfully synthesized micelles of ca. 100 nm of a hydrophilic [poly(oligoethylene glycol)] methyl ether methacrylate, POEGMA, allocated in the shell of the micelle – hydrophobic [poly(t-butyl acrylate)], PtBA, in the core of the micelle) copolymer following polymerization-induced self-assembly (PISA) approach. The end group of the POEGMA moiety was modified with a NTA (nitrile acetic acid) group in order to target His-tagged enzymes. With this strategy, they achieved a recyclable and robust biocatalysts based on horseradish peroxidase and esterase enzymes (Keller et al., 2017). Further, the composition of the polymer that prompt the assembly into micelles might have a positive effect on the activity and the stability of the biocatalyst. Indeed, it has been described that Pluronic F127-based micelles enhance the activity of organophosphate hydrolase (OPH) enzyme and its stability compared to the native version under broad conditions (room and high temperature, after multiple freeze/thaw treatments, lyophilization, and in the presence of organic solvents). This interesting effect can be attributed to a possible interaction of the hydrophobic polypropylene oxide block of the Pluronic F127 and the hydrophobic surface domains on the enzyme that are close to ligand pockets (Suthiwangcharoen and Nagarajan, 2014).

##### Dendrimers

Dendrimers (*ca.* 100–200 nm) are another interesting tool for enzyme immobilization. A dendrimer is a polymeric molecule composed of multiple branched monomers that emanate radially from a central hydrophobic core, giving rise to micelle-like behavior (Gupta et al., 2006) (Figure 5B). The multiple branches and the high density of functional end-groups of the dendrimers enable the high effectiveness of immobilization through covalent bonding. For example, polyester or poly(amidoamine) (PAMAM) dendrimers can be used as a convenient platform for glucose oxidase or lipase enzymes (Fan et al., 2017; Morshed et al., 2019). Dendrimer-enzyme hybrids, mostly PAMAM-based dendrimer particles, are also employed for delivery purposes in biomedicine. In the work of Liu C. et al. (2019), the enzyme immobilization is carried out through the phenylboronic acid (PBA) complex (i.e., cationic amine and imidazole groups on proteins *via* nitrogen-boronate complexation). This configuration leads to unprecedented efficiency for cytosolic delivery of proteins with different isoelectric points and sizes such as Cas9/sgRNA, trypsin, β-galactosidase, lysozyme, cytochrome C, horseradish peroxidase, and RNase A (Liu C. et al., 2019). Other examples in which PAMAM dendrimer scaffold is modified with dipicolylamine (DPA)/zinc (II) complex have demonstrated great efficiency in the delivery of enzymes, superior to that showed by commercial TransEx and PULSin delivery systems (Ren et al., 2020).

##### Nanoparticles

Inorganic nanomaterials, i.e., nanoparticles (50–200 nm), are usually combined with an interfacial capping layer composed of organic molecules, often polymers, in order to provide stability to the nanomaterial and, at the same time, to facilitate the localization of the enzyme on the surface (Figure 5C). Polymers such as acrylamide, cellulose, and chitosan are mostly used to tether enzymes through both non-covalent and covalent conjugation methods (Bilal and Iqbal, 2019a). With this strategy, the direct enzyme-nanoparticle interaction, which usually leads to a partial denaturation of the protein, is avoided. The presence of polymer, either as coatings or linkers, instead impart a favorable environment to the enzymes (Rodrigues et al., 2013). The main application of these biohybrids are focused on catalysis or biotransformations, as inorganic nanoparticles commonly help enhancing the catalytic activity and recyclability (Breger et al., 2015). Polymer coated magnetic nanoparticles, mostly composed of magnetite and maghemite, are known to be good platforms for catalysts. They show low toxicity, high enzyme loading capacity, and ease the recycling of the biocatalyst. As example, Ngo et al. (2012) fabricated a magnetic nanobiocatalyst by the conjugation of alcohol dehydrogenase (ADH) enzyme to poly(glycidyl methacrylate) magnetic nanoparticles (PGMA-MNPs) using glutaraldehyde (GA) as chemical crosslinker. The confined enzymes showed the same activity as the native ADH, with a reaction yield of the 97 and 99% of enantiomeric excess (ee, R) in 60 min. The system was easy to recycle, keeping the activity around 80% after 14 cycles of 20 min each (Ngo et al., 2012). Other enzymes, such as *O*-acetylserine sulfhydrylase (OASS), have been immobilized on PGMA-MNPs through Ni^2+^/His-tag affinity binding (Vahidi et al., 2016). Further, non-magnetic nanoparticles (e.g., metallic or silica) are also used (Zhang C. et al., 2018) as core material. There are recent and interesting examples of enzymes [e.g., *Candida antarctica* lipase B (CALB), *Candida antarctica* lipase A (CALA) and OPH], covalently bound to the polymeric shell of metal mesoporous nanoparticles. These systems enabled two-step one-pot dynamic kinetic resolution (DKR) of 1-phenylethylamine and a β-amino ester (ethyl 3-amino-3-phenylpropanoate) in organic solvents, and the degradation of organophosphate nerve agent (methyl parathion) in aqueous solution. In all cases high reaction yields (75% conversion) and enantiomeric excess (98% ee) were reached (Gao et al., 2020). Importantly, the nature and length of polymeric coats and linkers, as well as the size of the nanoparticles and the methodology used to tether the enzymes, have a direct effect on the activity and selectivity of the biocatalyst. As studied by the group of Prof. Manuel Ferrer, protein flexibility constrains can be modulated through cautious design of the material and the strength of the linkage between the enzyme and the polymer (Coscolín et al., 2018). Interestingly, they found that short and rigid polymeric linkers limit the flexibility and dynamics of the enzyme, reducing its activity with larger substrates.

##### Giant amphiphiles

Finally, another route for the fabrication of enzyme decorated polymer nanoparticles is based on the synthesis of the so-called “giant amphiphiles,” used effectively in sensing applications and heterogeneous catalysis. This method relies on the self-assembly of amphiphilic enzyme-polymer conjugates, being the enzyme the polar headgroup and the synthetic polymers the nonpolar tail of the macromolecule (Figure 5D). The conjugates act as giant surfactants that form protein covered nanoparticles in aqueous solutions in a relatively easy manner (Delaittre et al., 2009). This approach manages to control the morphology of the nanostructure and the orientation of the enzyme, and at the same time preserves the stability and the activity of the biocatalyst. This system demands the modification of the protein at only one single point. Therefore, affinity approaches (biotin-streptavidin binding, cofactor reconstitution) (Boerakker et al., 2006), or site-selective covalent polymer conjugation are used. As usual, the conjugation approach affects the stability of the micelle and the protein orientation (Huang and Olsen, 2016). In a recent example, enzyme-poly(*N*-(2-hydroxypropyl) methacrylate)) (PHPMA) conjugates were self-assembled through polymerization-induced coassembly (PICA) approach. The water-insoluble PHPMA was synthetized by ATRP using glucose oxidase (GOx)-Br and horseradish peroxidase (HRP)-Br macroinitiators. Thereafter, the GOx/HRP-PHPMA conjugates were assembled *in situ* into co-micelles during the polymerization reaction. The co-micelles showed 4.9-fold cascade activity enhancement compared to free enzymes, and were used for a fast glucose detection (Chiang et al., 2020). Other chemistries, i.e., CuAAC click chemistry, have lately been used for the generation of self-assembled bioconjugated nanoparticles (Bao et al., 2018).

##### Encapsulation of enzymes in the core of polymeric hollow structures

**FIGURE 6:**
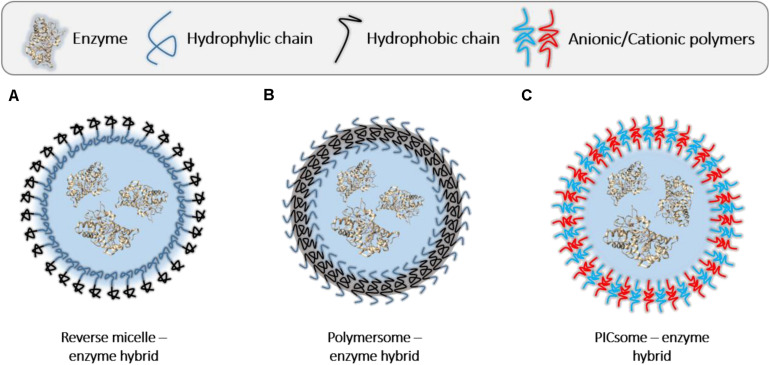
Assembled enzyme-polymer supramolecular hybrids in which the enzyme is confined in the inner cavity of the structures. **(A)** Reverse micelle-enzyme hybrid. **(B)** Polymersome-enzyme hybrid. **(C)** PICsome-enzyme hybrid.

In many cases, supramolecular polymeric structures are exploited to form enzymatic nanoreactors by filling the interior of the self-assembled polymers with guest enzymes (Delaittre et al., 2009) (Figure 6 and Table 3). Several polymeric architectures are being used for the encapsulation of enzymes: reverse micelles, polymersomes, PICsomes, and hydrogel nanoparticles. Although the catalytic activity can be altered by the confinement, mostly due to mass-transfer issues, this strategy can be interesting to protect them from the action of proteases and to avoid their aggregation.

##### Reverse micelles

Polymer-based reverse micelles (RMs) (10–50 nm) are spherical structures formed by surfactant aggregates (Figure 6A). The hydrophilic heads are oriented to the micelle core due to dipole–dipole and ion–dipole interaction, whereas the hydrophobic tails are in contact with the surrounding organic solvent. The low amount of water added to the solvent is confined near to the polar heads forming a small “water pool” that facilities the immobilization of the enzyme and the possibility to carry out reactions with hydrophobic substrates (Biasutti et al., 2008). The hydrophobic polymer shell makes the micelle stable in organic solvents and prevents the enzyme from denaturation. Cationic cetyltrimethyl-ammoniumbromide (CTAB), anionic bis(2-ethylhexyl) sulfosuccinate (AOT) and nonionic polyoxyethylene sorbitan trioleate (Tween 85) are the most used amphiphilic polymers (Chen H. et al., 2008). Interestingly, it has been proven that some enzymes such as, α-chymotrypsin (Moyano et al., 2010), lipases (Maiti et al., 2011) or horseradish peroxidase (Zhong et al., 2016) show enhanced catalytic properties when they are confined in reverse micelles. It has been proposed that this positive effect could be a consequence of the conformational changes suffered by the protein, the high concentration of substrates within the RM, or an altered hydration state of the active site of the enzyme (Moyano et al., 2010; Sintra et al., 2014; Gao et al., 2018). However, the principles behind the so-called enzyme “superactivity” have not been elucidated yet.

##### Polymersomes

Polymersomes (100–400 nm) are vesicle-like structures with a large size variability, which bilayer membrane is composed of amphiphilic block copolymers that mimic nature liposomes (Matoori and Leroux, 2020) (Figure 6B). The encapsulation of the enzymes inside the polymersomes occurs *in situ*, while the supramolecular arrangement of the block copolymers takes place. These polymeric structures are attractive for studying enzymatic reactions because they provide protection to the enzyme, localized in the core, from harsh environmental conditions and can confine the reaction within the nanospace, with the possibility to design multi-compartment systems (Klermund et al., 2017). Indeed, the confinement of enzymes in such small volumes can reduce their *K*_m_ and increase their *k*_cat_ (Chen et al., 2009). However, although this mechanism seems convenient from the perspective of the protein (there is no need of modification of the biomacromolecule), several issues are found. The assembly of the polymersomes in presence of the protein usually shows a low encapsulation efficiency and a high heterogeneity of the sample. Moreover, it is often difficult to control the permeability of the membrane of this sort of hybrids, essential for the diffusion of reaction substrates and products (Blackman et al., 2018). For overcoming these issues, porous or stimuli-responsive membranes are used, as well as membrane channel proteins (Klermund et al., 2017). Hydrophilic polymers such as poly(2-methyl-2-oxazoline) (PMOXA), poly(ethylene glycol) (PEG), poly(isocyano-L-alanine(2-thiophen-3-yl-ethyl)amide) (PIAT), and hydrophobic polydimethylsiloxane and polystyrene (PS) are among the most used blocks for the synthesis of polymersomes (Belluati et al., 2019). Regarding the employed enzymes, this system is suitable for peroxidases, oxidases, dehydrogenases, and catalases (Gaitzsch et al., 2016).

##### PICsomes

Hydrophobicity is not the only noncovalent interaction used for the synthesis of nanometric polymeric capsules. There are several works focused on the formation of enzyme-loaded polyion complex (PIC) vesicles (PICsomes) (Figure 6C), which are formed through the electrostatic interaction-mediated self-assembly between oppositely charged hydrophilic block copolymers (Anraku et al., 2010). The PICsome membrane is semipermeable and enables the uptake of the substrate of the enzyme and the release of products, while the enzymes are retained in the interior. Under mechanical stress, PICsomes tend to disassemble but, after the removal of perturbations, they have the ability to spontaneously reassemble, which is a good chance for re-encapsulating enzymes. Lately, PICsomes have been used for the successful immobilization of asparaginase (Sueyoshi et al., 2017) and β-Galactosidase (Anraku et al., 2016), with potential usefulness in delivery applications.

##### MOF-enzyme nanohybrids

**FIGURE 7:**
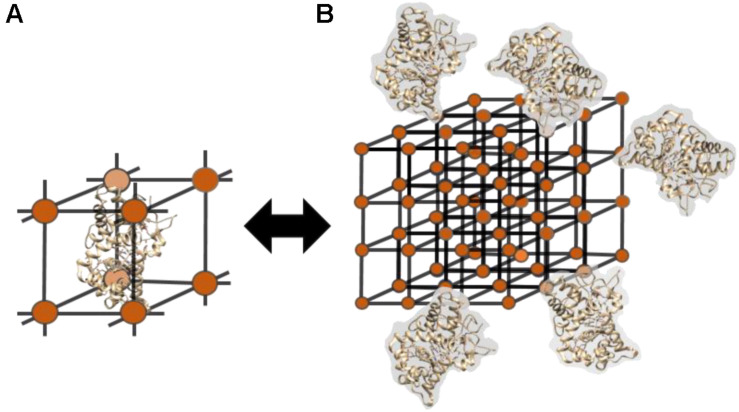
Enzyme-MOF hybrids can be synthesized either by the infiltration of the protein inside the pores of the material **(A)** or through the immobilization of the enzyme on the surface of the organic framework **(B)**.

Enzymes can be immobilized in the porous network of nanoscale coordination polymers (N), better known as polymer-based metal organic frameworks (MOFs) (500 nm–2 μm). NCPs are synthetized in presence of organic solvents and high temperatures and thereafter mixed with the enzyme in an aqueous phase to allow the enzyme infiltration into the pores (Chen et al., 2012) (Figure 7A). Alternatively, enzymes can be conjugated to the surface of the polymeric network by physical adsorption or covalent binding (Gkaniatsou et al., 2017; Liang et al., 2020) (Figure 7B). Finally, several methodologies such as coprecipitation (Lyu et al., 2014) or biomimetic biomineralization (Liang et al., 2016) allow the *in situ* incorporation of enzymes in the protective MOF structure. The selection of the most appropriate method for the synthesis of the hybrids must consider the synthesis conditions of the MOFs (usually not compatible with enzymes) and the pore size of the network (large enzymes might not fit in it).

Protein-NCP hybrids have been applied to a wide range of applications, such as sensing (Wang et al., 2016; Gao et al., 2017; Vinita et al., 2018; Wu et al., 2019; Xiong et al., 2020), microfluidic systems (Hu et al., 2020), removal of pollutants (Shen et al., 2019), photodynamic therapies (Cheng et al., 2016; Li et al., 2017; You et al., 2019; He et al., 2020), protein delivery (Chen et al., 2018; Yang et al., 2019; Zhang et al., 2019a; Alyami et al., 2020) or even for the optimization of the polymerase chain reaction (PCR) (Sun et al., 2020). Undoubtedly, the nature of the ligands and metals used for the synthesis of the MOFs has a direct impact on the final structure of the hybrid and eventually on the activity of the enzyme. In this regard, the porosity of the MOF is a key factor that can be tuned selectively in the synthesis procedure. MOFs synthesized with divalent metals such as Zn^2+^, Fe^2+^, and Cu^2+^ and imidazole-derived ligands, are the most employed for enzyme immobilization [see examples with catalase (Liang et al., 2019; Li et al., 2020), lipase (He H. et al., 2016; Pitzalis et al., 2018), HRP (Wu et al., 2017), cytochrome c (Zhang et al., 2017), β-galactosidase (Chen et al., 2018), and caspase-3 (CP-3) (Chen et al., 2018) collected in Table 3].

Finally, it is worth mentioning that also covalent organic frameworks (COFs) have currently been used as support materials for enzyme immobilization. In a very recent article, Li et al. (2020) achieved enzyme-COF capsules (co-immobilizing catalase and glucose oxidase by encapsulation). They used a MOF (ZIF-90 or Zeolitic Pyrimidine Framework, ZPF-2) structure as sacrificial template to make COF capsules. COFs provide an alternative with high porosity, stability, and readily engineered functionality to the formation of enzyme-polymer hybrids. The large porosity of COFs makes them suitable as platform for the enzyme, favoring the mass transfer of the substrate and the conformational freedom of the enzyme. Albeit this is a very recent approach applied to the field of enzyme-polymer hybrids, we do foresee the development of highly interesting enzyme-COF hybrids in the coming years due to the advantageous features that COF networks can provide to the hybrid system.

### Assembly of Enzyme-Polymer Hybrids Into Microstructures

**FIGURE 8:**
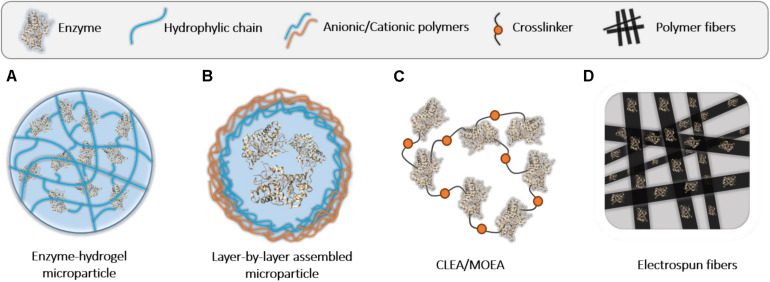
Schematic illustration of enzyme-polymer microhybrids described in this work. **(A)** Enzyme-hydrogel microparticles. **(B)** Microparticles or vesicles formed by LbL assembly. **(C)** CLEAs or MOEAs. **(D)** Enzyme-polymer fiber hybrids.

The embedment of enzymes into micro- or macroscopic polymeric structures has several benefits. Usually, the larger the hybrid, the better the recycling potential is. This fact improves substantially the overall economy of the process for a particular biocatalytic procedure. Compared to macrostructures, microscaled polymers usually show lower diffusion issues and fairly good upscaling capacity, which makes them one of the best options for the design of biocatalytic bioreactors (see collated examples in Table 4). In this section, we focus our attention on enzyme hybrids fabricated with micrometric polymeric hydrogels, layer-by-layer wise assembled enzyme microparticles, crosslinking polymers, and electrospun polymeric fibers (illustrations in Figure 8).

**TABLE 4:** Summary of the most relevant examples of enzyme-polymer microhybrids.

Structure	Non-enzymatic component	Enzyme	Immobilization method	Hybrid size	Characteristics (regarding activity)	Application	References
Hydrogels	PEG^3^	Lac^15^	Entrapment	Macro	Six cycles (100%)	Polymerization hydroquinone	Zhang et al., 2020
	PEG, dithiothreitol (DTT)	ALP^16^, GOx^17^	Covalent binding (Click chem)	9–32 μm	[Enzyme] dependent increase	Model for tissue engineering, drug delivery, and biosensing	Jivan et al., 2016
	PNIPAM^4^	Cellulase	Entrapment	450 nm	85% retained after six cycles	Bagasse saccharification in [EMIM]OAc^29^	Zhou et al., 2019
	PEGDA^5^/Dextran	CAT^18^	Entrapment	20 μm	52% of the original after UV exposure	Micromotor	Keller et al., 2018
	Alginate	LOx^19^/CAT	Entrapment	11 μm	20 cycles (100%)	Lactate sensing	Biswas et al., 2017
LbL^1^ microparticle	Poly dextran/poly-L-arginine	ASN^20^	Encapsulation (LbL)	1–2 μm	Slightly enhanced thermostability	Model for therapy	Karamitros et al., 2013
CLEPCs^2^	Pluronic F127	OPH^21^	Adsorption	NA*	High activity. MeOH resistant	Bioremediation of OPs^30^	Kim et al., 2014
	Pluronic F127	OPH	Covalent (GA^24^)	*ca.* 600 nm	Activity 2.2-fold vs. CLEAs. Activity 2.5-fold with detergents	Bioremediation of OPs	Cheng et al., 2018
	Imidazole-PAA^6^	GOx	Encapsulation and affinity binding	400–900 nm	Slight decrease and high thermostability	Model for biocatalysis	Rodriguez-Abetxuko et al., 2019a
	Agar, chitosan, dextran and gum arabic	α-amylase	Covalent (biopolymers oxidized)	NA*	Higher activity retention	Model of biocompatible CLEAs^31^	Nadar et al., 2016
Polymer fibers	PAN^7^/MMT^8^/GO^9^	Lac	Adsorption	*ca.* d. 200 nm	39% of removal. GO increased stability	Bioremediation	Wang et al., 2014
	PMMA^10^/Fe_3_O_4_	Lac	Covalent binding (EDC^25^/NHS^26^), entrapment	*ca.* d. 500 nm	High activity retained upon 40 days store	Bioremediation	Zdarta et al., 2019a
	PLCL^11^	Lac	Entrapment, adsorption	*ca.* d. 500 nm	Adsorbed lower efficiency. *K*_m_^app^ads < *K*_m_^app^entr^27^.	Bioremediation	Zdarta et al., 2019b
	PAN/PEDOT^12^	GOx	Covalent binding (GA)	*ca.* d. 800 nm	Lowest LOD 2.9 μM. Lowest *K*_m_^app^ 0.057 μM.	Amperometric biosensor of glucose	Çetin and Camurlu, 2018
	PS^13^/PSMA^14^	bCA^22^	Covalent binding (GA)	*ca.* d. 5 μm	Activity preserved. Stability of *ca.* 800 days	Bioremediation	Jun et al., 2020
	Chitosan-gelatine	HRP^23^	Covalent binding (GA)	*ca.* d. 80 nm	Activity preserved. LOD^28^ 0.05 mM	Electrochemical biosensor of H_2_O_2_	Teepoo et al., 2017

*^∗^Not available; ^1^layer-by-Layer; ^2^cross-linked enzyme-polymer conjugates; ^3^polyethylene glycol; ^4^poly(N-isopropylacrylamide); ^5^poly(ethylene glycol) diacrylate; ^6^poly(acrylic acid); ^7^poly(acrylonitrile); ^8^montmorillonite; ^9^graphene oxide; ^10^poly(methyl methacrylate); ^11^poly(L-lactide-co-ε-caprolactone); ^12^poly(3,4-ethylenedioxythiophene); ^13^polystyrene; ^14^poly(styrene-co-maleic anhydride); ^15^laccase; ^16^alkaline phosphatase; ^17^glucose oxidase; ^18^catalase; ^19^lipoxygenase; ^20^asparaginase; ^21^organophosphate hydrolase; ^22^bovine carbon anhydrase; ^23^horseradish peroxidase; ^24^glutaraldehyde; ^25^1-Ethyl-3-(3-dimethylaminopropyl)carbodiimide; ^26^N-hydroxysuccinimide; ^27^apparent Michaelis constant of adsorption and entrapment; ^28^limit of detection; ^29^1-ethyl-3-methyllimidazolium acetate; ^30^organophosphorus; ^31^cross-linked enzyme aggregates.*

#### Enzyme Hydrogel Microhybrids

Polymeric hydrogels are three-dimensional polymeric network structures composed by natural or synthetic polymers with the ability to soak up high amounts of water and swell. The water absorption capability arises from hydrophilic groups (e.g., -OH, -CONH_2_, and -SO_3_H) in the structure, and the swelling behavior is related to the crosslinked structure, built by covalent and non-covalent bonds. Importantly, this architecture provides stability to the biomolecules, as well as high enzyme loading capacities (Hamidi et al., 2008) (Figure 8A). Hydrogels with a wide range of sizes can be targeted, ranging from 10 nm of the SENs, above described, to macroscopic enzyme-loaded sponges (Zhang et al., 2020), which are not in the focus of this review. The catalytic reaction rates of the latter are lower due to diffusion impairment caused by the polymer network. In contrast, the substrate/product diffusion path in enzyme-hydrogel microhybrids is shortened and, as consequence, the substrate is more rapidly exposed to the enzyme. Among the natural polymers, alginate, dextran, gelatin, chitosan, and agarose are the most studied ones due to their non-toxicity, biocompatibility, biodegradability, renewability, and the availability of seating numerous reactive sites (Beldengrün et al., 2018; Bilal and Iqbal, 2019b). The most employed synthetic polymers for the fabrication of hydrogel microparticles are poly(vinyl alcohol) (PVA), polyethyleneimine (PEI), polyvinylpyrrolidone (PVP), polyethyleneglycol (PEG), and poly(*N*-isopropylacrylamide) (PNIPAAM) (Pachioni-Vasconcelos et al., 2016). The synthesis procedure of the hybrids is very diverse. Proteins can be decorated with exogeneous reactive groups that will eventually be used to anchor the preformed polymeric network (Ji et al., 2016). For this aim, the use of click chemistry (e.g., sequential thiol-ene and bio-orthogonal tetrazine-norbornene click reactions) to control the synthesis of the hybrids can be an appreciated tool (Jivan et al., 2016). Alternatively, the direct mixture of preformed hydrogel microparticles with the enzyme solution has led to interesting microscopic hydrogels in which enzymes remain entrapped through weak interactions (Zhou et al., 2019). Recently, enzyme loaded hydrogel microparticles have been employed for the development of biosensors and enzyme-driven micromotors (Biswas et al., 2017; Keller et al., 2018).

#### Layer-by-Layer Wise Assembled Enzyme-Polymer Microhybrids

Layer-by-layer (LbL) deposition of ionic polymers is a commonly used methodology for the formation of micrometric hybrid biocatalysts. The fabrication consists in oppositely charged polyelectrolytes that assemble layer-wise on solid supports, usually a charged surface or an inorganic sacrificial template. This technique is conveniently explored for enzyme immobilization due to the mild conditions utilized in the assembly procedure, that help preserving enzyme folding and hence their stability (Figure 8B). It enables the on-demand design of the composition, thickness, charge, and permeability of the formed particles or films. Different micrometric designs for enzyme immobilization have been synthetized by LbL, such as microspheres, capsules, and hollow capsules (Sakr and Borchard, 2013). The most utilized ionic polymers are polystyrene sulfonate (PSS), poly(allylamine hydrochloride) (PAH), poly(ethylene imine) (PEI), and alginate (polysaccharide), which are usually combined with enzymes (typically catalase, glucose oxidase, α-chemotrypsin or β-glucosidase). As mentioned above, inorganic particles (mainly made of calcium carbonate) with embedded enzymes are mainly used as sacrificial templates (Yu et al., 2005; Karamitros et al., 2013; Parakhonskiy et al., 2014). However, in a very interesting example, Caruso and coworkers developed a smooth and productive enzyme encapsulation strategy that consisted of the formation of the LbL assembly on the surface of enzyme crystals, skipping the use of templates. With this approach, the enzymes are hydrated after the assembly process and remain trapped inside the capsule (Caruso et al., 2000).

#### Cross-Linked Enzyme-Polymer Conjugates

The synthesis of cross-linked enzyme aggregates (CLEAs) is a widely employed enzyme immobilization method. Basically, enzymes are covalently connected by a cross-linking agent. The most used cross-linker is glutaraldehyde (GA) due to its high conversion efficiencies, low cost, and high market availability (Velasco-Lozano et al., 2016). However, the density of accessible lysine residues is sometimes low for an effective cross-linking. In this case, additives such as polymers or protein feeders are commonly introduced, e.g., PEIs (López-Gallego et al., 2005), PEI-sulfate dextran (Wilson et al., 2004), dodecyl aldehyde (Guimarães et al., 2018), bovine serum albumin (BSA) (Torabizadeh et al., 2014; Amaral-Fonseca et al., 2018), egg albumin (Šulek et al., 2011), poly-lysine (Yamaguchi et al., 2011), and soy protein isolate (SPI) (Araujo-Silva et al., 2018). However, several issues such as its high toxicity and reactivity have been found with the use of GA as crosslinking agent. For this reason, (bio)polymers have been attempted in the last years, giving rise to cross-linked enzyme-polymer conjugates (CLEPCs), e.g., dextran-polyaldehyde (DP) (Mateo et al., 2004; Sahutoglu and Akgul, 2015), gum Arabic (Jin et al., 2019), chitosan (Nadar et al., 2016), and ethylene glycol-bis[succinic acid *N*-hydroxysuccinimide] (EG-NHS) (Rehman et al., 2016). Recently, our group has developed a methodology for the cross-linking of single enzyme nanogels, triggered by metal-imidazole coordination, resulting the so-called metal-organic enzyme aggregates (MOEAs). High enzyme loadings and low diffusional issues of MOEAs make them extraordinary candidates for biocatalysis applications (Rodriguez-Abetxuko et al., 2019a) (see collated examples with main results in Table 4).

#### Enzyme-Polymer Fiber Hybrids

Electrospun nanofibers are alternative polymeric structures that make use of electrospinning technology (Cleeton et al., 2019) to form large fibers. The most important features of the materials produced by electrospinning are the high degree of porosity (provided by the features of the polymer), the biocompatibility, and the high number of functional groups that can be displayed on the surface (mainly –NH_2_ and –OH). Thus, this method significantly increases the enzyme loads in comparison with conventional supports. On the last two decades, this technique has been employed to immobilize diverse (bio)molecules, including enzymes, in applications such as biosensors (Teepoo et al., 2017), pollution control (Jun et al., 2020), or bioreactors (Sakai et al., 2008) (Figure 8D). So far, two methodologies are followed for the fabrication of the enzyme-nanofiber hybrids: entrapment (*in situ* immobilization) and surface attachment (adsorption or covalent binding). Despite the drawbacks of immobilization by adsorption, i.e., reliance on the composition of the enzyme and protein leakage, it is an excellent approach that enables the increase of the activity and reusability of the enzyme (Wang Q. et al., 2013; Wang et al., 2006, 2014). On the other hand, covalent binding offers an improvement of the enzyme-support stability to a larger extent (Li et al., 2007; Feng et al., 2012, 2013; Zhang et al., 2014). In a recent study, Zdarta et al. (2019a) compared both methods of immobilization using polymeric fibers. They employed poly(L-lactic acid)-co-poly(ε-caprolactone) (PLCL) electrospun nanofibers to immobilize laccase by either entrapment or adsorption approach for the biodegradation of hazardous pollutants from wastewaters. They observed an increase of the affinity (lower *K*_m_^app^) of the adsorbed enzyme toward the substrate. Yet, the efficiency of the removal of pollutants by this hybrid was lower, mainly due to the deactivation of the enzyme and its leakage from the support. When covalent binding and entrapment methods were compared in terms of stability, they observed also significant differences. Both showed a significant enhancement with respect of free enzyme, which lost half of its activity after 20 days. However, while the hybrid fabricated through the covalent approach showed the 75% of the initial activity after 40 days of storage, the entrapped enzyme conserved 90% of the activity (Zdarta et al., 2019a). Other polymeric fibers such as poly(vinyl alcohol) (PVA) (Dinçer and Telefoncu, 2007), polyamide 6 (PVA/PA6) (Feng et al., 2014), polyacrylonitrile (PAN) (Wang et al., 2014), amidoxime polyacrylonitrile (AOPAN) (Zhang et al., 2014), and poly(3,4-ethylenedioxythiophene) (PEDOT)/PAN (Çetin and Camurlu, 2018) have been explored as supports for enzymes.

### Assembly of Enzyme-Polymer Hybrids Into Macrostructures

**FIGURE 9:**
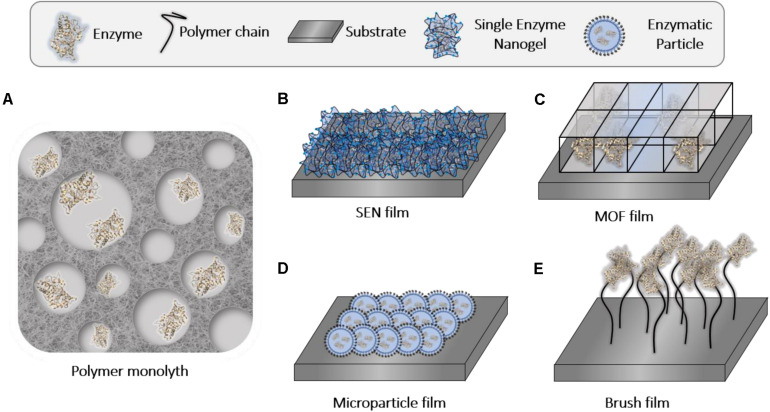
Schematic illustration of enzyme-polymer monoliths and enzyme-polymer film types formed following different approaches mentioned in this work: **(A)** polymer monoliths; **(B)** assembly of SENs; **(C)** assembly of MOFs; **(D)** assembly of microparticles; and **(E)** attachment to polymer brushes.

Finally, in this last section, we highlight the use of macroassembled polymers into monoliths and continuous films as platforms for enzyme immobilization (Figure 9 and Table 5). The size of these materials provides the hybrids with good mechanical properties, expanding their applicability.

**TABLE 5:** Summary of the most relevant examples of polymer-enzymes macrostructures.

Structure	Non-enzymatic moiety	Enzyme	Immobilization method	Characteristics (regarding activity)	Application	References
Monolith	Poly(BMA-MAA-EDMA)^2^	Trypsin	Covalent binding (thiol-ene)	80% activity upon 5 cycles;864 times faster digestion	IMERs^37^	Zhao et al., 2020
	Methacrylate Convective Interaction Media (CIM) disks	Cytochrome P450	Affinity (biotin-avidine)	Reduction hydroxilation up to 75% in presence of drug	IMERs	Nicoli et al., 2008
	Ethylenediamine (EDA) CIM disks	Arginase	Covalent (GA^27^)	*K*_m_^app^ *ca* 14 mM^31^	IMERs	André et al., 2011
	pDM/P/Au^3^	Trypsin	Adsorption	*K*_m_^app^13.9 mg/mL; *V*_max_^app^16.1 μg/mL/min^32^	IMERs	Zhao et al., 2019
	TRIM^4^	Trypsin	Covalent binding (thiol-ene)	79.41% sequence coverage of BSA^33^	IMERs	Fan et al., 2020
CP^1^ film	PTTBA^5^/AuNPs	AChE^19^/ChO^20^	Covalent	LOD^34^ 0.6 nM	Electrochemical biosensor of Ach^38^	Akhtar et al., 2017
	PTTCA^6^	GluOx^21^	Covalent (EDC^28^)	LOD 0.1 μM	Biosensor Glu	Rahman et al., 2005
	MWCNT^7^/PTTCA/AuNPs	MP^22^, CAT^23^, SOD^24^	Covalent (EDC-NHS^29^)	LOD 4.3 nM	Electrochemical biosensor of NO	Abdelwahab et al., 2010
	PEI^8^/CNT^9^	GOx^25^/CAT	Covalent	*K*_m_^app^ 2.6, 4.3 and 4.1 mM (GOx, CAT, and GOx/CAT) MPD^35^ 180.8 μW?cm^–2^	EBFC^39^/GBFC^40^	Christwardana et al., 2017
Polymer film	CdTe(QDs^10^)/PDDA^11^	GOx, Tyrosinase	Adsorption	LOD 10 mM (catechol);LOD 5 mM (glucose)	Fluorescent biosensor of glucose and catechol	Yuan et al., 2012
	PPE^12^/poly(aniline)	β-lactamase	Adsorption (LbL^30^ technique)	High and fast activity	Fluorescent biosensor of penicillin G	Vázquez et al., 2007
	CTPR^13^ proteins	CAT	Covalent (GA)	Activity preserved. Reusability	Energy device	Sánchez-deAlcázar et al., 2019
	HEAA^14^, MBAAm^15^, Vim^16^	GOx, β-Glu^26^	Affinity binding	LR^36^ 0.1–1.5 × 10^–3^ m. 400-fold increase in isopropanol vs. H_2_O	Electrochemical biosensor and biotransformation	Rodriguez-Abetxuko et al., 2019b
	Cu^2+^/Hemin	GOx	*In situ* encapsulation	LOD 2.73 μM; sensitivity 22.77 μA mM^–1^ cm^–2^	Biosensor of glucose	He J. et al., 2016
Polymer Brush	PSBMA^17^, PEGMA^18^	Lipase	Covalent (NHS) + Non-covalent interaction	>100-fold increase	Model for biocatalysis and biosensing	Weltz et al., 2019

*^1^Conducting polymers; ^2^butyl methacrylate-α-methacrylic acid-ethylene glycol dimethacrylate; ^3^cryogel composite; ^4^trimethylolpropane trimethacrylate; ^ 5^2,2:5,2- terthiophene-3-(p-benzoic acid); ^6^Poly-5,2′:5′,2′′-terthiophene-3′-carboxylic acid; ^7^multi-walled carbon nanotubes; ^8^polyethylenimine; ^9^carbon nanotubes; ^10^quantum dots; ^11^poly(dimethyldiallylammonium chloride); ^ 12^polyphenyl ether; ^13^consensus tetratricopeptide repeat protein; ^14^hydroethylacrylamide; ^15^N,N′-methylenebisacrylamide; ^16^vinyl imidazole; ^17^poly(sulfobetaine methacrylate); ^18^poly(poly(ethylene glycol) methacrylate); ^19^acetylcholinesterase; ^20^cholinesterase; ^21^glutamate oxidase; ^22^microperoxidase; ^23^catalase; ^24^superoxide dismutase; ^25^glucose oxidase; ^26^β-glucosidase; ^27^glutaraldehyde; ^28^1-Ethyl-3-(3-dimethylaminopropyl)carbodiimide; ^29^N-hydroxysuccinimide; ^30^layer-by-layer; ^31^apparent Michaelis constant; ^32^maximam velocity; ^33^bovine serum albumin; ^34^limit of detection; ^35^maximum power density; ^36^linear range; ^37^immobilized monolith-enzyme reactor; ^38^acetylcholine; ^39^enzymatic biofuel cell; ^ 40^glucose biofuel cell.*

#### Polymer Monoliths-Enzyme Hybrids

Polymer monoliths are very interesting macrostructures developed in 1990s, mainly based on methacrylates, acrylates, and styrenes. The polymerization is performed in templates such as rod polymer for chromatographic columns (Watanabe et al., 2009; Li et al., 2010; Duan et al., 2020), disks (Lin et al., 2020), and microfluidic channels (Knob et al., 2016; Parker et al., 2019; Zhu et al., 2019). Monoliths can be functionalized with different groups such as epoxy, aldehyde or alkene groups (Masini and Svec, 2017). These supports have been used for the stabilization of enzymes in both aqueous media and organic solvents (Lv et al., 2014; Wen et al., 2016a; Luo et al., 2019). However, the main drawback of polymer monoliths relies on the large size of their pores, which results in low enzyme loads. Fortunately, this limitation can be circumvented by grafting a polymer layer on the surface of the macropores of monoliths (Peterson et al., 2003; Li et al., 2014; Wen et al., 2016b).

Enzyme-monolith hybrids (Figure 9A) have a relevant impact in microfluidic applications (Logan et al., 2007; Krenkova et al., 2009; Meller et al., 2017; Cheng et al., 2019) and, in less extension, biosensors (Luo et al., 2019), and HPLC (Girelli and Mattei, 2005). However, its preferential and successful application is as a bio-reactor platform for proteomic analysis (Peterson et al., 2002; Geiser et al., 2008; Sproß and Sinz, 2010; Calleri et al., 2011; Liang et al., 2011; Naldi et al., 2017; Han et al., 2019; Zhang et al., 2019). On a very recent example (Zhao et al., 2020), it has been shown a straightforward method for the fabrication of an immobilized monolith-enzymatic reactor (IMER) for proteome analysis (coupled to ESI-MS technique). The immobilization process was carried out by “thiol-ene” click chemistry. The thiol groups of the trypsin enzyme reacted with alkene groups of a polymer monolith fabricated with ethylene glycol dimethacrylate (EDMA) to form a thioether. Enzyme turned out to be very active with a *K*_m_^app^ of 2.1 mM, quite similar to that measured for the free enzyme (*K*_m_ 1.4 mM). In addition, the enzyme maintained the activity up to 80% after five cycles. In the same line, they extended their IMER design using trimethylolpropane trimethacrylate (TRIM), instead of EDMA, increasing the anchoring points of the support. Thus, they raised the enzymatic load and hence the specific activity of the hybrid (Fan et al., 2020; Wei et al., 2020) (details in Table 5). Also, IMERs have been applied for the immobilization of other enzymes involved in other pathologies such as arginase in cardiovascular diseases (André et al., 2011), acetylcholinesterase (AChE) in the treatment of Alzheimer disease (De Simone et al., 2019) or in metabolism studies using the cytochrome P450 enzyme (Nicoli et al., 2008).

#### Enzyme-Polymer Films

The application of enzyme-polymer films are rather relevant in distinct scientific areas and applications such as biosensors (Vázquez et al., 2007; Yuan et al., 2012), muti-enzymatic cascade reactions (Sharma et al., 2013; Farrugia et al., 2017), or enzymatic biofuel cells (EBFCs) (Chung et al., 2016; Christwardana et al., 2017). In particular, polymer-enzyme films-based biosensors have been widely studied as alternative to the traditional chemical methodologies. The assembly of enzyme-polymer hybrids in film fashion can be performed by simple deposition or drop casting (Rodriguez-Abetxuko et al., 2019b; Sánchez-deAlcázar et al., 2019), by spin-coating (Chen B. et al., 2008), by layer-by-layer approaches (Scodeller et al., 2014; Zhang et al., 2019b), by electrospinning (Henke et al., 2020), by dip-coating (Marquitan et al., 2020) or by Langmuir–Blodget technique (Qian et al., 2002). Smaller enzyme-polymer hybrids, e.g., MOFs, particles or SENs, (Dong et al., 2018; Liu et al., 2018; Wang et al., 2018; Sureka et al., 2019; Henke et al., 2020), can be thereby deposited in continuous films to form responsive biocoatings (Figures 9B,C). For example, single enzyme nanogels of glucose oxidase are able to assemble into ordered and highly stable films by means of coordination polymers and divalent metals (Rodriguez-Abetxuko et al., 2019b). Also, Cu^2+^-based tyrosinase MOFs can be used to fabricate a bisphenol A biosensor (Wang et al., 2015; Lu et al., 2016).

##### Use of conductive polymers

Conducting polymers (CPs) have attracted the attention because they exhibit interesting properties for the development of biosensors (Marcus and Sutin, 1985). CPs have high electrical conductivity, low ionization potential, high electronic affinities, as well as optical properties. The most used systems are based on polyacetylene, polyaniline (PANI), polypyrrole (Ppy), polythiophene, poly (*p*-phenylene), and poly (phenylene vinyl-ene). Successful CPs-based enzyme biosensors have been fabricated for the detection of biological molecules such as glutamic acid (Rahman et al., 2005; Kergoat et al., 2014), acetylcholine (Fenoy et al., 2020), and soluble gasses as NO (Abdelwahab et al., 2010), all of them associated to particular diseases. As an example, the adsorption of acetylcholinesterase (AChE) and choline oxidase (ChO) enzymes on a film of Fe_2_O_3_NPs/poly(3,4-ethylenedioxythiophene (PEDOT))-reduced graphene oxide (rGO)/modified fluorine doped tin oxide (FTO) electrode reported a detection limit of acetyl choline as low as 4 nM. The proposed sensor was applied to determine acetyl choline in serum samples from healthy and Alzheimer’s patients. Since acetylcholine concentration is slightly lower in Alzheimer’s patients than in healthy individuals (5.0–7.8 nM vs. 8.2–11.3 nM, respectively), this system is capable to discriminate both populations (Chauhan et al., 2017). In order to overcome the drawbacks related to enzyme immobilization (mainly related to protein leakage) and enhance the sensitivity, Akhtar et al. (2017) covalently co-immobilized both enzymes (AChE and ChO). With this approach, the sensitivity was reduced significantly, reporting a detection limit of *ca.* 0.6 nM and a usability of 60 days maintaining the 91% of the sensitivity. Similarly, highly sensitive CP-based glucose biosensors, which are relevant for patients suffering from Diabetes’s disease, have also been developed using glucose oxidase (GOx) enzyme. The use of GOx-loaded PtNPs-PEDOT microspheres has shown the best reported sensor of PEDOT up to date (Piro et al., 2001; Nien et al., 2006; David et al., 2018; Liu et al., 2018).

##### Use of polymeric brushes

Aiming at modifying the surface of materials, i.e., electrodes or particles, the use of polymeric brushes can be an alternative to film deposition. Polymer brushes are long and flexible polymer chains, attached to surfaces or other polymer chains, with high grafting density (Figure 9E). The chemistry, the molecular weight, and the grafting density can be wisely tailored. These parameters may have an effect on the structure and hence the activity of the enzyme. Furthermore, polymeric brushes act as an extension of the surface into a third dimension, granting a high and tunable density of functional groups (epoxide, carboxylic acid, hydroxyl, aldehyde, and amine groups) that can be eventually used for the conjugation of enzymes (Jiang and Xu, 2013). Several publications have pointed out that hydrophilic or zwitterionic polymer brushes create favorable microenvironments for catalysis (Weltz et al., 2019). However, there is still some work to do in the fabrication of efficient enzyme-polymer brush systems. The amount of reactive groups within the polymer brushes should be optimized for each case, as the high density of those leads to a reduced degree of conformational freedom of the enzyme, which is generally translated to lower bioactivities (Jiang and Xu, 2013).

## Conclusion and Outlook

In this review, we have brought together different approaches to enhance the stabilization or increase the activity of enzymes using tunable polymers as non-catalytic supporting materials. With different examples, we have demonstrated that the polymer is usually more than a mere support. Polymers can be used to protect enzymes from denaturation, to increase the bioactivity through beneficial interactions, to concentrate the enzymes in small volumes, to confine the enzymes in favorable environments, or to ease the recyclability of the enzyme. These hybrids are built up as a result of the careful selection of each component, i.e., enzyme and polymer, and the strategy utilized for their combination. The current synthesis methods have facilitated the control over the amount, position of the conjugation-site, and orientation of the immobilized enzymes, as well as the molecular architecture of the resultant hybrid. Furthermore, we have shown that nanohybrids are suitable for nanomedicine applications, mainly due to an easier transport, biocompatibility, and more adequate delivery inside the body. Moreover, the high enzyme/polymer ratio content showed by enzyme-polymer conjugates (EPCs), single enzyme nanogels (SENs), giant amphiphiles or reverse micelles make them attractive for novel applications. On the other hand, bigger structures, such as polymer monoliths or films, have a more relevant impact as reactors or biosensors, respectively. All in all, new, refined, and sophisticated hybrid structures with novel functionalities are sought. However, the reusability and storage of the immobilized enzymes is still the limiting factor for a cost-effective applicability and commercialization of these systems. Finally, we anticipate the emergence of new and appealing approaches in the field of enzyme-polymer hybrids as novel polymeric architectures and synthesis methodologies are being developed.

## Author Contributions

AR-A and DS revised the literature and wrote the manuscript. PM designed the figures. AB supervised the work, designed the figures, and wrote the manuscript.

## Conflict of Interest

The authors declare that the research was conducted in the absence of any commercial or financial relationships that could be construed as a potential conflict of interest.

## References

[B1] Abdelwahab A. A. Koh W. C. A. Noh H.-B. Shim Y.-B. (2010). A selective nitric oxide nanocomposite biosensor based on direct electron transfer of microperoxidase: removal of interferences by co-immobilized enzymes. *Biosens. Bioelectron.* 26 1080–1086. 10.1016/j.bios.2010.08.070 20864332

[B2] Adrio J. L. Demain A. L. (2014). Microbial enzymes: tools for biotechnological processes. *Biomolecules* 4 117–139. 10.3390/biom4010117 24970208 PMC4030981

[B3] Akhtar M. H. Hussain K. K. Gurudatt N. G. Shim Y. B. (2017). Detection of Ca2+-induced acetylcholine released from leukemic T-cells using an amperometric microfluidic sensor. *Biosens. Bioelectron.* 98 364–370. 10.1016/j.bios.2017.07.003 28704785

[B4] Alyami M. Z. Alsaiari S. K. Li Y. Qutub S. S. Aleisa F. A. Sougrat R. (2020). Cell-Type-specific CRISPR/Cas9 delivery by biomimetic metal organic frameworks. *J. Am. Chem. Soc.* 142 1715–1720. 10.1021/jacs.9b11638 31931564

[B5] Amaral-Fonseca M. Kopp W. Giordano R. Fernández-Lafuente R. Tardioli P. (2018). Preparation of magnetic cross-linked amyloglucosidase aggregates: solving some activity problems. *Catalysts* 8:496 10.3390/catal8110496

[B6] André C. Herlem G. Gharbi T. Guillaume Y. C. (2011). A new arginase enzymatic reactor: development and application for the research of plant-derived inhibitors. *J. Pharm. Biomed. Anal.* 55 48–53. 10.1016/j.jpba.2011.01.003 21310573

[B7] Anraku Y. Kishimura A. Kamiya M. Tanaka S. Nomoto T. Toh K. (2016). Systemically injectable enzyme-loaded polyion complex vesicles as in vivo nanoreactors functioning in tumors. *Angew. Chem. Int. Edn.* 55 560–565. 10.1002/anie.201508339 26629778

[B8] Anraku Y. Kishimura A. Oba M. Yamasaki Y. Kataoka K. (2010). Spontaneous formation of nanosized unilamellar polyion complex vesicles with tunable size and properties. *J. Am. Chem. Soc.* 132 1631–1636. 10.1021/ja908350e 20078126

[B9] Araujo-Silva R. Mafra A. Rojas M. Kopp W. Giordano R. Fernandez-Lafuente R. (2018). Maltose production using starch from cassava bagasse catalyzed by cross-linked β-amylase aggregates. *Catalysts* 8:170 10.3390/catal8040170

[B10] Arnold F. H. (2018). Directed Evolution: Bringing New Chemistry to Life. *Angew. Chem. Int. Edn.* 57 4143–4148. 10.1002/anie.201708408 29064156 PMC5901037

[B11] Assarsson A. Pastoriza-Santos I. Cabaleiro-Lago C. (2014). Inactivation and adsorption of human carbonic anhydrase II by nanoparticles. *Langmuir* 30 9448–9456. 10.1021/la501413r 24999988

[B12] Averick S. Mehl R. A. Das S. R. Matyjaszewski K. (2015). Well-defined biohybrids using reversible-deactivation radical polymerization procedures. *J. Control. Release* 205 45–57. 10.1016/j.jconrel.2014.11.030 25483427

[B13] Baker S. L. Munasinghe A. Murata H. Lin P. Matyjaszewski K. Colina C. M. (2018). Intramolecular interactions of conjugated polymers mimic molecular chaperones to stabilize protein-polymer conjugates. *Biomacromolecules* 19 3798–3813. 10.1021/acs.biomac.8b00927 30086223

[B14] Baker S. L. Murata H. Kaupbayeva B. Tasbolat A. Matyjaszewski K. Russell A. J. (2019). Charge-preserving atom transfer radical polymerization initiator rescues the lost function of negatively charged protein-polymer conjugates. *Biomacromolecules* 20 2392–2405. 10.1021/acs.biomac.9b00379 31079461

[B15] Balcão V. M. Vila M. M. D. C. (2015). Structural and functional stabilization of protein entities: State-of-the-art. *Adv. Drug Deliv. Rev.* 93 25–41. 10.1016/j.addr.2014.10.005 25312675

[B16] Bao C. Yin Y. Zhang Q. (2018). Synthesis and assembly of laccase-polymer giant amphiphiles by self-catalyzed CuAAC click chemistry. *Biomacromolecules* 19 1539–1551. 10.1021/acs.biomac.8b00087 29562131

[B17] Barbosa O. Ortiz C. Berenguer-Murcia A. Torres R. Rodrigues R. C. Fernandez-Lafuente R. (2015). Strategies for the one-step immobilization-purification of enzymes as industrial biocatalysts. *Biotechnol. Adv.* 33 435–456. 10.1016/j.biotechadv.2015.03.006 25777494

[B18] Bayramoglu G. Yilmaz M. Arica M. Y. (2010). Reversible immobilization of laccase to poly(4-vinylpyridine) grafted and Cu(II) chelated magnetic beads: biodegradation of reactive dyes. *Bioresour. Technol.* 101 6615–6621. 10.1016/j.biortech.2010.03.088 20388589

[B19] Beldengrün Y. Aragon J. Prazeres S. F. Montalvo G. Miras J. Esquena J. (2018). Gelatin/maltodextrin water-in-water (W/W) emulsions for the preparation of cross-linked enzyme-loaded microgels. *Langmuir* 34 9731–9743. 10.1021/acs.langmuir.8b01599 29954182

[B20] Belluati A. Craciun I. Meyer C. E. Rigo S. Palivan C. G. (2019). Enzymatic reactions in polymeric compartments: nanotechnology meets nature. *Curr. Opin. Biotechnol.* 60 53–62. 10.1016/j.copbio.2018.12.011 30708278

[B21] Beloqui A. Baur S. Trouillet V. Welle A. Madsen J. Bastmeyer M. (2016). Single-molecule encapsulation: a straightforward route to highly stable and printable enzymes. *Small* 12 1716–1722. 10.1002/smll.201503405 26849308

[B22] Beloqui A. Cortajarena A. L. (2020). Protein-based functional hybrid bionanomaterials by bottom-up approaches. *Curr. Opin. Struct. Biol.* 63 74–81. 10.1016/j.sbi.2020.04.005 32485564

[B23] Beloqui A. Kobitski A. Y. Nienhaus G. U. Delaittre G. (2018). A simple route to highly active single-enzyme nanogels. *Chem. Sci.* 9 1006–1013. 10.1039/C7SC04438K 29675147 PMC5883864

[B24] Biasutti M. A. Abuin E. B. Silber J. J. Correa N. M. Lissi E. A. (2008). Kinetics of reactions catalyzed by enzymes in solutions of surfactants. *Adv. Colloid Interf. Sci.* 136 1–24. 10.1016/j.cis.2007.07.001 17706582

[B25] Bilal M. Iqbal H. M. N. (2019a). Chemical, physical, and biological coordination: an interplay between materials and enzymes as potential platforms for immobilization. *Coord. Chem. Rev.* 388 1–23. 10.1016/j.ccr.2019.02.024

[B26] Bilal M. Iqbal H. M. N. (2019b). Naturally-derived biopolymers: potential platforms for enzyme immobilization. *Int. J. Biol. Macromol.* 130 462–482. 10.1016/j.ijbiomac.2019.02.152 30825566

[B27] Biswas A. Bornhoeft L. R. Banerjee S. You Y. H. McShane M. J. (2017). Composite hydrogels containing bioactive microreactors for optical enzymatic lactate sensing. *ACS Sens.* 2 1584–1588. 10.1021/acssensors.7b00648 29043796

[B28] Biswas A. Joo K. Liu J. Zhao M. Fan G. Wang P. (2011). Endoprotease-mediated intracellular protein delivery using nanocapsules. *ACS Nano* 5 1385–1394. 10.1021/nn1031005 21268592

[B29] Blackman L. D. Varlas S. Arno M. C. Houston Z. H. Fletcher N. L. Thurecht K. J. (2018). Confinement of therapeutic enzymes in selectively permeable polymer vesicles by polymerization-induced self-assembly (PISA) reduces antibody binding and proteolytic susceptibility. *ACS Cent. Sci.* 4 718–723. 10.1021/acscentsci.8b00168 29974067 PMC6026775

[B30] Boerakker M. J. Botterhuis N. E. Bomans P. H. H. Frederik P. M. Meijer E. M. Nolte R. J. M. (2006). Aggregation behavior of giant amphiphiles prepared by cofactor reconstitution. *Chem. A Eur. J.* 12 6071–6080. 10.1002/chem.200600089 16688714

[B31] Bontempo D. Maynard H. D. (2005). Streptavidin as a macroinitiator for polymerization: in situ protein-polymer conjugate formation. *J. Am. Chem. Soc.* 127 6508–6509. 10.1021/ja042230 15869252

[B32] Bosio V. E. Islan G. A. Martínez Y. N. Durán N. Castro G. R. (2016). Nanodevices for the immobilization of therapeutic enzymes. *Crit. Rev. Biotechnol.* 36 447–464. 10.3109/07388551.2014.990414 25641329

[B33] Boyer C. Bulmus V. Davis T. P. (2009). Efficient usage of thiocarbonates for both the production and the biofunctionalization of polymers. *Macromol. Rapid Commun.* 30 493–497. 10.1002/marc.200800708 21706630

[B34] Boyer C. Bulmus V. Liu J. Davis T. P. Stenzel M. H. Barner-Kowollik C. (2007). Well-defined protein-polymer conjugates via in situ RAFT polymerization. *J. Am. Chem. Soc.* 129 7145–7154. 10.1021/ja070956a 17500523

[B35] Breger J. C. Ancona M. G. Walper S. A. Oh E. Susumu K. Stewart M. H. (2015). Phosphotriesterase kinetic efficiency. *ACS Nano* 9 8491–8503. 10.1021/acsnano.5b03459 26230391

[B36] Calleri E. Temporini C. Gasparrini F. Simone P. Villani C. Ciogli A. (2011). Immobilized trypsin on epoxy organic monoliths with modulated hydrophilicity: novel bioreactors useful for protein analysis by liquid chromatography coupled to tandem mass spectrometry. *J. Chromatogr. A* 1218 8937–8945. 10.1016/j.chroma.2011.05.059 21679957

[B37] Canalle L. A. Löwik D. W. P. M. Van Hest J. C. M. (2010). Polypeptide-polymer bioconjugates. *Chem. Soc. Rev.* 39 329–353. 10.1039/b807871h 20023856

[B38] Care A. Petroll K. Gibson E. S. Y. Bergquist P. L. Sunna A. (2017). Solid-binding peptides for immobilisation of thermostable enzymes to hydrolyse biomass polysaccharides. *Biotechnol. Biofuels* 10:29. 10.1186/s13068-017-0715-2 28184244 PMC5289021

[B39] Carmali S. Murata H. Cummings C. Matyjaszewski K. Russell A. J. (2017). Polymer-based protein engineering: synthesis and characterization of armored, high graft density polymer-protein conjugates. *Methods Enzymol.* 590 347–380. 10.1016/bs.mie.2016.12.005 28411645

[B40] Caruso F. Trau D. Möhwald H. Renneberg R. (2000). Enzyme encapsulation in layer-by-layer engineered polymer multilayer capsules. *Langmuir* 16 1485–1488. 10.1021/la991161n

[B41] Çetin M. Z. Camurlu P. (2018). An amperometric glucose biosensor based on PEDOT nanofibers. *RSC Adv.* 8 19724–19731. 10.1039/c8ra01385c PMC9080667 35541002

[B42] Chapman R. Stenzel M. H. (2019). All wrapped up: stabilization of enzymes within single enzyme nanoparticles. *J. Am. Chem. Soc.* 141 2754–2769. 10.1021/jacs.8b10338 30621398

[B43] Chauhan N. Chawla S. Pundir C. S. Jain U. (2017). An electrochemical sensor for detection of neurotransmitter-acetylcholine using metal nanoparticles, 2D material and conducting polymer modified electrode. *Biosens. Bioelectron.* 89 377–383. 10.1016/j.bios.2016.06.047 27342368

[B44] Chen B. Pernodet N. Rafailovich M. H. Bakhtina A. Gross R. A. (2008). Protein immobilization on epoxy-activated thin polymer films: effect of surface wettability and enzyme loading. *Langmuir* 24 13457–13464. 10.1021/la8019952 18991420

[B45] Chen H. Liu L. H. Wang L. S. Ching C. B. Yu H. W. Yang Y. Y. (2008). Thermally responsive reversed micelles for immobilization of enzymes. *Adv. Funct. Mater.* 18 95–102. 10.1002/adfm.200600452

[B46] Chen G. Abdeen A. A. Wang Y. Shahi P. K. Robertson S. Xie R. (2019). A biodegradable nanocapsule delivers a Cas9 ribonucleoprotein complex for in vivo genome editing. *Nat. Nanotechnol.* 14 974–980. 10.1038/s41565-019-0539-2 31501532 PMC6778035

[B47] Chen Q. Schönherr H. Vancso G. J. (2009). Block-copolymer vesicles as nanoreactors for enzymatic reactions. *Small* 5 1436–1445. 10.1002/smll.200801455 19283796

[B48] Chen T. T. Yi J. T. Zhao Y. Y. Chu X. (2018). Biomineralized metal-organic framework nanoparticles enable intracellular delivery and endo-lysosomal release of native active proteins. *J. Am. Chem. Soc.* 140 9912–9920. 10.1021/jacs.8b04457 30008215

[B49] Chen Y. Lykourinou V. Vetromile C. Hoang T. Ming L. J. Larsen R. W. (2012). How can proteins enter the interior of a MOF? investigation of cytochrome c translocation into a MOF consisting of mesoporous cages with microporous windows. *J. Am. Chem. Soc.* 134 13188–13191. 10.1021/ja305144x 22862180

[B50] Cheng H. Zhao Y. L. Luo X. J. Xu D. S. Cao X. Xu J. H. (2018). Cross-linked enzyme-polymer conjugates with excellent stability and detergent-enhanced activity for efficient organophosphate degradation. *Bioresour. Bioprocess.* 5:49 10.1186/s40643-018-0236-2

[B51] Cheng H. Zhu J. Y. Li S. Y. Zeng J. Y. Lei Q. Chen K. W. (2016). An O_2_ self-sufficient biomimetic nanoplatform for highly specific and efficient photodynamic therapy. *Adv. Funct. Mater.* 26 7847–7860. 10.1002/adfm.201603212

[B52] Cheng M. Wang R. Zhang B. Mao Z. Chen Z. (2019). Rapid proteolytic digestion and peptide separation using monolithic enzyme microreactor coupled with capillary electrophoresis. *J. Pharm. Biomed. Anal.* 165 129–134. 10.1016/j.jpba.2018.11.063 30529826

[B53] Chiang C. W. Liu X. Sun J. Guo J. Tao L. Gao W. (2020). Polymerization-induced coassembly of enzyme-polymer conjugates into comicelles with tunable and enhanced cascade activity. *Nano Lett.* 20 1383–1387. 10.1021/acs.nanolett.9b04959 31891508

[B54] Christwardana M. Chung Y. Kwon Y. (2017). Co-immobilization of glucose oxidase and catalase for enhancing the performance of a membraneless glucose biofuel cell operated under physiological conditions. *Nanoscale* 9 1993–2002. 10.1039/c6nr09103b 28106225

[B55] Chung Y. Ahn Y. Christwardana M. Kim H. Kwon Y. (2016). Development of a glucose oxidase-based biocatalyst adopting both physical entrapment and crosslinking, and its use in biofuel cells. *Nanoscale* 8 9201–9210. 10.1039/C6NR00902F 27074999

[B56] Cleeton C. Keirouz A. Chen X. Radacsi N. (2019). Electrospun nanofibers for drug delivery and biosensing. *ACS Biomater. Sci. Eng.* 5 4183–4205. 10.1021/acsbiomaterials.9b00853 33417777

[B57] Cobo I. Li M. Sumerlin B. S. Perrier S. (2015). Smart hybrid materials by conjugation of responsive polymers to biomacromolecules. *Nat. Mater.* 14 143–149. 10.1038/nmat4106 25401924

[B58] Coscolín C. Beloqui A. Martínez-Martínez M. Bargiela R. Santiago G. Blanco R. M. (2018). Controlled manipulation of enzyme specificity through immobilization-induced flexibility constraints. *Appl. Catal. A Gen.* 565 59–67. 10.1016/j.apcata.2018.08.003

[B59] Coulet P. R. Carlsson J. Porath J. (1981). Immobilization of enzymes on metal-chelate regenerable carriers. *Biotechnol. Bioeng.* 23 663–668. 10.1002/bit.260230316

[B60] Cuomo F. Ceglie A. De Leonardis A. Lopez F. (2019). Polymer capsules for enzymatic catalysis in confined environments. *Catalysts* 9 1–18. 10.3390/catal9010001

[B61] Dai G. Hu J. Zhao X. Wang P. (2017). A colorimetric paper sensor for lactate assay using a cellulose-Binding recombinant enzyme. *Sens. Actuat. B Chem.* 238 138–144. 10.1016/j.snb.2016.07.008

[B62] Daunert S. Bachas L. G. Schauer-Vukasinovic V. Gregory K. J. Schrift G. Deo S. (2007). Calmodulin-mediated reversible immobilization of enzymes. *Colloids Surf. B Biointerf.* 58 20–27. 10.1016/j.colsurfb.2006.10.020 17276043

[B63] David M. Barsan M. M. Brett C. M. A. Florescu M. (2018). Improved glucose label-free biosensor with layer-by-layer architecture and conducting polymer poly(3,4-ethylenedioxythiophene). *Sens. Actuat. B Chem.* 255 3227–3234. 10.1016/j.snb.2017.09.149

[B64] De Simone A. Naldi M. Bartolini M. Davani L. Andrisano V. (2019). Immobilized enzyme reactors: an overview of applications in drug discovery from 2008 to 2018. *Chromatographia* 82 425–441. 10.1007/s10337-018-3663-5

[B65] Dedisch S. Wiens A. Davari M. D. Soder D. Rodriguez-Emmenegger C. Jakob F. (2020). Matter- tag?: a universal immobilization platform for enzymes on polymers, metals, and silicon-based materials. *Biotechnol. Bioeng.* 117 49–61. 10.1002/bit.27181 31549734

[B66] Delaittre G. Reynhout I. C. Cornelissen J. J. L. M. Nolte R. J. M. (2009). Cascade reactions in an all-enzyme nanoreactor. *Chem. A Eur. J.* 15 12600–12603. 10.1002/chem.200902063 19859924

[B67] Dinçer A. Telefoncu A. (2007). Improving the stability of cellulase by immobilization on modified polyvinyl alcohol coated chitosan beads. *J. Mol. Catal. B Enzym.* 45 10–14. 10.1016/j.molcatb.2006.10.005

[B68] Dong S. Peng L. Wei W. Huang T. (2018). Three MOF-templated carbon nanocomposites for potential platforms of enzyme immobilization with improved electrochemical performance. *ACS Appl. Mater. Interf.* 10 14665–14672. 10.1021/acsami.8b00702 29620852

[B69] Duan R. Sun L. Yang H. Y. Ma Y. R. Deng X. Y. Peng C. (2020). Preparation of phenyl-boronic acid polymeric monolith by initiator-free ring-opening polymerization for microextraction of sulfonamides prior to their determination by ultra-performance liquid chromatography-tandem mass spectrometry. *J. Chromatogr. A* 1609:460510. 10.1016/j.chroma.2019.460510 31515077

[B70] Fan P. R. Zhao X. Wei Z. H. Huang Y. P. Liu Z. S. (2020). Robust immobilized enzyme reactor based on trimethylolpropane trimethacrylate organic monolithic matrix through “thiol-ene” click reaction. *Eur. Polym. J.* 124:109456 10.1016/j.eurpolymj.2019.109456

[B71] Fan Y. Su F. Li K. Ke C. Yan Y. (2017). Carbon nanotube filled with magnetic iron oxide and modified with polyamidoamine dendrimers for immobilizing lipase toward application in biodiesel production. *Nat. Publ. Gr.* 2017 1–13. 10.1038/srep45643 28358395 PMC5372472

[B72] Fang Y. Zhang A. Li S. Sproviero M. Xu M. Q. (2019). Enzyme immobilization for solid-phase catalysis. *Catalysts* 9 12–15. 10.3390/catal9090732

[B73] Farrugia T. Perriman A. W. Sharma K. P. Mann S. (2017). Multi-enzyme cascade reactions using protein-polymer surfactant self-standing films. *Chem. Commun.* 53 2094–2097. 10.1039/c6cc09809f 28124039

[B74] Feng Q. Tang B. Wei Q. Hou D. Bi S. Wei A. (2012). Preparation of a Cu(II)-PVA/PA6 composite nanofibrous membrane for enzyme immobilization. *Int. J. Mol. Sci.* 13 12734–12746. 10.3390/ijms131012734 23202922 PMC3497296

[B75] Feng Q. Wang Q. Tang B. Wei A. Wang X. Wei Q. (2013). Immobilization of catalases on amidoxime polyacrylonitrile nanofibrous membranes. *Polym. Int.* 62 251–256. 10.1002/pi.4293

[B76] Feng Q. Zhao Y. Wei A. Li C. Wei Q. Fong H. (2014). Immobilization of catalase on electrospun PVA/PA6-Cu(II) nanofibrous membrane for the development of efficient and reusable enzyme membrane reactor. *Environ. Sci. Technol.* 48 10390–10397. 10.1021/es501845u 25093534

[B77] Fenoy G. E. Marmisollé W. A. Azzaroni O. Knoll W. (2020). Acetylcholine biosensor based on the electrochemical functionalization of graphene field-effect transistors. *Biosens. Bioelectron.* 148:111796. 10.1016/j.bios.2019.111796 31665672

[B78] Gaitzsch J. Huang X. Voit B. (2016). Engineering functional polymer capsules toward smart nanoreactors. *Chem. Rev.* 116 1053–1093. 10.1021/acs.chemrev.5b00241 26322859

[B79] Gao J. Wang C. Tan H. (2017). Lanthanide/nucleotide coordination polymers: an excellent host platform for encapsulating enzymes and fluorescent nanoparticles to enhance ratiometric sensing. *J. Mater. Chem. B* 5 7692–7700. 10.1039/c7tb02049j 32264370

[B80] Gao J. Zhao B. Wang M. Serrano M. A. C. Zhuang J. Ray M. (2018). Supramolecular assemblies for transporting proteins across an immiscible solvent interface. *J. Am. Chem. Soc.* 140 2421–2425. 10.1021/jacs.7b13245 29431433 PMC6174083

[B81] Gao S. Wang Z. Ma L. Liu Y. Gao J. Jiang Y. (2020). Mesoporous core-shell nanostructures bridging metal and biocatalyst for highly efficient cascade reactions. *ACS Catal.* 10 1375–1380. 10.1021/acscatal.9b04877

[B82] Gauthier M. A. Klok H. A. (2010). Polymer-protein conjugates: an enzymatic activity perspective. *Polym. Chem.* 1 1352–1373. 10.1039/c0py90001j

[B83] Ge J. Lu D. Wang J. Liu Z. (2009). Lipase nanogel catalyzed transesterification in anhydrous dimethyl sulfoxide. *Biomacromolecules* 10 1612–1618. 10.1021/bm900205r 19361213

[B84] Ge J. Lu D. Wang J. Yan M. Lu Y. Liu Z. (2008). Molecular fundamentals of enzyme nanogels. *J. Phys. Chem. B* 112 14319–14324. 10.1021/jp8053923 18939792

[B85] Geiser L. Eeltink S. Svec F. Fréchet J. M. J. (2008). In-line system containing porous polymer monoliths for protein digestion with immobilized pepsin, peptide preconcentration and nano-liquid chromatography separation coupled to electrospray ionization mass spectroscopy. *J. Chromatogr. A* 1188 88–96. 10.1016/j.chroma.2008.02.075 18342870 PMC2435401

[B86] Girelli A. M. Mattei E. (2005). Application of immobilized enzyme reactor in on-line high performance liquid chromatography?: a review. *Science* 819 3–16. 10.1016/j.jchromb.2005.01.031 15797515

[B87] Gkaniatsou E. Sicard C. Ricoux R. Mahy J. P. Steunou N. Serre C. (2017). Metal-organic frameworks: a novel host platform for enzymatic catalysis and detection. *Mater. Horizons* 4 55–63. 10.1039/c6mh00312e

[B88] Gu Z. Yan M. Hu B. Joo K. L. Biswas A. Huang Y. (2009). Protein nanocapsule weaved with enzymatically degradable polymerie network. *Nano Lett.* 9 4533–4538. 10.1021/nl902935b 19995089

[B89] Guimarães J. Giordano R. Fernandez-Lafuente R. Tardioli P. (2018). Evaluation of strategies to produce highly porous cross-linked aggregates of porcine pancreas lipase with magnetic properties. *Molecules* 23:2993. 10.3390/molecules23112993 30453506 PMC6278321

[B90] Guisán J. M. (1988). Aldehyde-agarose gels as activated supports for immobilization-stabilization of enzymes. *Enzyme Microb. Technol.* 10 375–382. 10.1016/0141-0229(88)90018-X

[B91] Gupta U. Agashe H. B. Asthana A. Jain N. K. (2006). Dendrimers: novel polymeric nanoarchitectures for solubility enhancement. *Biomacromolecules* 7 649–658. 10.1021/bm050802s 16529394

[B92] Hamidi M. Azadi A. Rafiei P. (2008). Hydrogel nanoparticles in drug delivery. *Adv. Drug Deliv. Rev.* 60 1638–1649. 10.1016/j.addr.2008.08.002 18840488

[B93] Han X. Xie Y. Wu Q. Wu S. (2019). The effect of monolith properties on the digestion performance of monolith-based immobilized enzyme microreactor. *J. Chromatogr. Sci.* 57 116–121. 10.1093/chromsci/bmy091 30272129

[B94] He H. Han H. Shi H. Tian Y. Sun F. Song Y. (2016). Construction of thermophilic lipase-embedded metal-organic frameworks via biomimetic mineralization: a biocatalyst for ester hydrolysis and kinetic resolution. *ACS Appl. Mater. Interf.* 8 24517–24524. 10.1021/acsami.6b05538 27580160

[B95] He J. Yang H. Zhang Y. Yu J. Miao L. Song Y. (2016). Smart nanocomposites of cu-hemin metal-organic frameworks for electrochemical glucose biosensing. *Sci. Rep.* 6:36637. 10.1038/srep36637 27811998 PMC5095656

[B96] He L. Ni Q. Mu J. Fan W. Liu L. Wang Z. (2020). Solvent-assisted self-assembly of a metal-organic framework based biocatalyst for cascade reaction driven photodynamic therapy. *J. Am. Chem. Soc.* 142 6822–6832. 10.1021/jacs.0c02497 32196319

[B97] Henke P. Dolanskı J. Kubát P. Mosinger J. (2020). Multifunctional photosensitizing and biotinylated polystyrene nanofiber membranes/composites for binding of biologically active compounds. *ACS Appl. Mater. Interf.* 12 18792–18802. 10.1021/acsami.9b23104 32216378

[B98] Hermanson G. T. (2008). *Bioconjugate Techniques.* Amsterdam: Elsevier.

[B99] Hiep Nguyen H. Kim M. (2017). An overview of techniques in enzyme immobilization. *Appl. Sci. Converg. Technol.* 26 157–163. 10.5757/ASCT.2017.26.6.157

[B100] Hola K. Markova Z. Zoppellaro G. Tucek J. Zboril R. (2015). Tailored functionalization of iron oxide nanoparticles for MRI, drug delivery, magnetic separation and immobilization of biosubstances. *Biotechnol. Adv.* 33 1162–1176. 10.1016/j.biotechadv.2015.02.003 25689073

[B101] Hu C. Bai Y. Hou M. Wang Y. Wang L. Cao X. (2020). Defect-induced activity enhancement of enzyme-encapsulated metal-organic frameworks revealed in microfluidic gradient mixing synthesis. *Sci. Adv.* 6:eaax5785. 10.1126/sciadv.aax5785 32064336 PMC6989138

[B102] Huang A. Olsen B. D. (2016). Self-assembly of differently shaped protein-polymer conjugates through modification of the bioconjugation site. *Macromol. Rapid Commun.* 37 1268–1274. 10.1002/marc.201500744 27322114

[B103] Jesionowski T. Zdarta J. Krajewska B. (2014). Enzyme immobilization by adsorption: a review. *Adsorption* 20 801–821. 10.1007/s10450-014-9623-y

[B104] Ji X. Liu J. Liu L. Zhao H. (2016). Enzyme-polymer hybrid nanogels fabricated by thiol-disulfide exchange reaction. *Coll. Surf. B Biointerf.* 148 41–48. 10.1016/j.colsurfb.2016.08.043 27591569

[B105] Jiang H. Xu F. J. (2013). Biomolecule-functionalized polymer brushes. *Chem. Soc. Rev.* 42 3394–3426. 10.1039/c2cs35453e 23348574

[B106] Jin W. Xu Y. Yu X. W. (2019). Preparation of lipase cross-linked enzyme aggregates in octyl-modified mesocellular foams. *Int. J. Biol. Macromol.* 130 342–347. 10.1016/j.ijbiomac.2019.02.154 30825565

[B107] Jivan F. Yegappan R. Pearce H. Carrow J. K. McShane M. Gaharwar A. K. (2016). Sequential thiol-ene and tetrazine click reactions for the polymerization and functionalization of hydrogel microparticles. *Biomacromolecules* 17 3516–3523. 10.1021/acs.biomac.6b00990 27656910

[B108] Jun S. H. Yang J. Jeon H. Kim H. S. Pack S. P. Jin E. (2020). Stabilized and immobilized carbonic anhydrase on electrospun nanofibers for enzymatic CO_2_ conversion and utilization in expedited microalgal growth. *Environ. Sci. Technol.* 54 1223–1231. 10.1021/acs.est.9b05284 31899628

[B109] Jung S. Kwon I. (2016). Expansion of bioorthogonal chemistries towards site-specific polymer-protein conjugation. *Polym. Chem.* 7 4584–4598. 10.1039/c6py00856a

[B110] Karamitros C. S. Yashchenok A. M. Möhwald H. Skirtach A. G. Konrad M. (2013). Preserving catalytic activity and enhancing biochemical stability of the therapeutic enzyme asparaginase by biocompatible multilayered polyelectrolyte microcapsules. *Biomacromolecules* 14 4398–4406. 10.1021/bm401341k 24144040

[B111] Keefe A. J. Jiang S. (2012). Poly(zwitterionic)protein conjugates offer increased stability without sacrificing binding affinity or bioactivity. *Nat. Chem.* 4 59–63. 10.1038/nchem.1213 22169873 PMC4059762

[B112] Keller D. Beloqui A. Martínez-Martínez M. Ferrer M. Delaittre G. (2017). Nitrilotriacetic amine-functionalized polymeric core-shell nanoparticles as enzyme immobilization supports. *Biomacromolecules* 18 2777–2788. 10.1021/acs.biomac.7b00677 28731680

[B113] Keller S. Teora S. P. Hu G. X. Nijemeisland M. Wilson D. A. (2018). High-throughput design of biocompatible enzyme-based hydrogel microparticles with autonomous movement. *Angew. Chem. Int. Edn.* 57 9814–9817. 10.1002/anie.201805661 29917309

[B114] Kergoat L. Piro B. Simon D. T. Pham M. C. Noël V. Berggren M. (2014). Detection of glutamate and acetylcholine with organic electrochemical transistors based on conducting polymer/platinum nanoparticle composites. *Adv. Mater.* 26 5658–5664. 10.1002/adma.201401608 24924118

[B115] Kim K. T. Cornelissen J. J. L. M. Nolte R. J. M. Van Hest J. C. M. (2009). A polymersome nanoreactor with controllable permeability induced by stimuli-responsive block copolymers. *Adv. Mater.* 21 2787–2791. 10.1002/adma.200900300

[B116] Kim M. Gkikas M. Huang A. Kang J. W. Suthiwangcharoen N. Nagarajan R. (2014). Enhanced activity and stability of organophosphorus hydrolase via interaction with an amphiphilic polymer. *Chem. Commun.* 50 5345–5348. 10.1039/c3cc47675h 24558645 PMC4059822

[B117] Klermund L. Poschenrieder S. T. Castiglione K. (2017). Biocatalysis in polymersomes: improving multienzyme cascades with incompatible reaction steps by compartmentalization. *ACS Catal.* 7 3900–3904. 10.1021/acscatal.7b00776

[B118] Knob R. Sahore V. Sonker M. Woolley A. T. (2016). Advances in monoliths and related porous materials for microfluidics. *Biomicrofluidics* 10:032901 10.1063/1.4948507 PMC4859832 27190564

[B119] Ko J. H. Maynard H. D. (2018). A guide to maximizing the therapeutic potential of protein-polymer conjugates by rational design. *Chem. Soc. Rev.* 47 8998–9014. 10.1039/c8cs00606g 30443654 PMC6322549

[B120] Koenig M. Bittrich E. König U. Rajeev B. L. Müller M. Eichhorn K. J. (2016). Adsorption of enzymes to stimuli-responsive polymer brushes: influence of brush conformation on adsorbed amount and biocatalytic activity. *Coll. Surf. B Biointerf.* 146 737–745. 10.1016/j.colsurfb.2016.07.015 27447452

[B121] Kovaliov M. Cheng C. Cheng B. Averick S. (2018). Grafting-from lipase: utilization of a common amino acid residue as a new grafting site. *Polym. Chem.* 9 4651–4659. 10.1039/c8py01026a

[B122] Kowsari M. Motallebi M. Zamani M. (2014). Protein engineering of chit42 towards improvement of chitinase and antifungal activities. *Curr. Microbiol.* 68 495–502. 10.1007/s00284-013-0494-3 24322404

[B123] Krenkova J. Lacher N. A. Svec F. (2009). Highly effcient enzyme reactors containing trypsin and endoproteinase lysc immobilized on porous polymer monolith coupled to ms suitable for analysis of antibodies. *Anal. Chem.* 81 2004–2012. 10.1021/ac8026564 19186936

[B124] Krishna O. D. Kiick K. L. (2010). Protein- and peptide-modified synthetic polymeric biomaterials. *Biopolymers* 94 32–48. 10.1002/bip.21333 20091878 PMC4437713

[B125] Labus K. Gancarz I. Bryjak J. (2012). Immobilization of laccase and tyrosinase on untreated and plasma-treated cellulosic and polyamide membranes. *Mater. Sci. Eng. C* 32 228–235. 10.1016/j.msec.2011.10.023

[B126] Li M. Qiao S. Zheng Y. Andaloussi Y. H. Li X. Zhang Z. (2020). Fabricating covalent organic framework capsules with commodious microenvironment for enzymes. *J. Am. Chem. Soc.* 142 6675–6681. 10.1021/jacs.0c00285 32197569

[B127] Li N. Zheng W. Shen Y. Qi L. Li Y. Qiao J. (2014). Preparation of a novel polymer monolith with functional polymer brushes by two-step atom-transfer radical polymerization for trypsin immobilization. *J. Sep. Sci.* 37 3411–3417. 10.1002/jssc.201400794 25196221

[B128] Li S. F. Chen J. P. Wu W. T. (2007). Electrospun polyacrylonitrile nanofibrous membranes for lipase immobilization. *J. Mol. Catal. B Enzym.* 47 117–124. 10.1016/j.molcatb.2007.04.010

[B129] Li S. Y. Cheng H. Xie B. R. Qiu W. X. Zeng J. Y. Li C. X. (2017). Cancer cell membrane camouflaged cascade bioreactor for cancer targeted starvation and photodynamic therapy. *ACS Nano* 11 7006–7018. 10.1021/acsnano.7b02533 28665106

[B130] Li Y. Dennis Tolley H. Lee M. L. (2010). Monoliths from poly(ethylene glycol) diacrylate and dimethacrylate for capillary hydrophobic interaction chromatography of proteins. *J. Chromatogr. A* 1217 4934–4945. 10.1016/j.chroma.2010.05.048 20576269

[B131] Liang K. Coghlan C. J. Bell S. G. Doonan C. Falcaro P. (2016). Enzyme encapsulation in zeolitic imidazolate frameworks: a comparison between controlled co-precipitation and biomimetic mineralisation. *Chem. Commun.* 52 473–476. 10.1039/c5cc07577g 26548587

[B132] Liang S. Wu X.-L. Xiong J. Zong M.-H. Lou W.-Y. (2020). Metal-organic frameworks as novel matrices for efficient enzyme immobilization: an update review. *Coord. Chem. Rev.* 406:213149 10.1016/j.ccr.2019.213149

[B133] Liang W. Xu H. Carraro F. Maddigan N. K. Li Q. Bell S. G. (2019). Enhanced activity of enzymes encapsulated in hydrophilic metal-organic frameworks. *J. Am. Chem. Soc.* 141 2348–2355. 10.1021/jacs.8b10302 30636404

[B134] Liang Y. Tao D. Ma J. Sun L. Liang Z. Zhang L. (2011). Hydrophilic monolith based immobilized enzyme reactors in capillary and on microchip for high-throughput proteomic analysis. *J. Chromatogr. A* 1218 2898–2905. 10.1016/j.chroma.2011.02.073 21450299

[B135] Lin Q. Tang M. Ke C. (2020). Thermo-responsive 3D-printed polyrotaxane monolith. *Polym. Chem.* 11 304–308. 10.1039/c9py01510h

[B136] Liu C. Wan T. Wang H. Zhang S. Ping Y. Cheng Y. (2019). A boronic acid-rich dendrimer with robust and unprecedented efficiency for cytosolic protein delivery and CRISPR-Cas9 gene editing. *Sci. Adv.* 5:eaaw8922. 10.1126/sciadv.aaw8922 31206027 PMC6561739

[B137] Liu Q. Xun G. Feng Y. (2019). The state-of-the-art strategies of protein engineering for enzyme stabilization. *Biotechnol. Adv.* 37 530–537. 10.1016/j.biotechadv.2018.10.011 31138425

[B138] Liu Y. Turner A. P. F. Zhao M. Mak W. C. (2018). Processable enzyme-hybrid conductive polymer composites for electrochemical biosensing. *Biosens. Bioelectron.* 100 374–381. 10.1016/j.bios.2017.09.021 28946109

[B139] Logan T. C. Clark D. S. Stachowiak T. B. Svec F. Fréchet J. M. J. (2007). Photopatterning enzymes on polymer monoliths in microfluidic devices for steady-state kinetic analysis and spatially separated multi-enzyme reactions. *Anal. Chem.* 79 6592–6598. 10.1021/ac070705k 17658765

[B140] López-Gallego F. Acebrón I. Mancheño J. M. Raja S. Lillo M. P. Guisán Seijas J. M. (2012). Directed, strong, and reversible immobilization of proteins tagged with a β-trefoil lectin domain: a simple method to immobilize biomolecules on plain agarose matrixes. *Bioconjug. Chem.* 23 565–573. 10.1021/bc2006237 22372708

[B141] López-Gallego F. Betancor L. Hidalgo A. Alonso N. Fernández-Lafuente R. Guisán J. M. (2005). Co-aggregation of enzymes and polyethyleneimine: a simple method to prepare stable and immobilized derivatives of glutaryl acylase. *Biomacromolecules* 6 1839–1842. 10.1021/bm050088e 16004417

[B142] Lu X. Wang X. Wu L. Wu L. Dhanjai J. Fu L. (2016). Response characteristics of bisphenols on a metal-organic framework-based tyrosinase nanosensor. *ACS Appl. Mater. Interfaces* 8 16533–16539. 10.1021/acsami.6b05008 27281291

[B143] Luo J. Ma L. Svec F. Tan T. Lv Y. (2019). Reversible two-enzyme coimmobilization on ph-responsive imprinted monolith for glucose detection. *Biotechnol. J.* 14:1900028. 10.1002/biot.201900028 31116006

[B144] Lv Y. Lin Z. Tan T. Svec F. (2014). Preparation of reusable bioreactors using reversible immobilization of enzyme on monolithic porous polymer support with attached gold nanoparticles. *Biotechnol. Bioeng.* 111 50–58. 10.1002/bit.25005 23860941

[B145] Lyu F. Zhang Y. Zare R. N. Ge J. Liu Z. (2014). One-pot synthesis of protein-embedded metal-organic frameworks with enhanced biological activities. *Nano Lett.* 14 5761–5765. 10.1021/nl5026419 25211437

[B146] Ma S. Li Y. Ma C. Wang Y. Ou J. Ye M. (2019). Challenges and advances in the fabrication of monolithic bioseparation materials and their applications in proteomics research. *Adv. Mater.* 31 1–27. 10.1002/adma.201902023 31502719

[B147] Maiti S. Ghosh M. Das P. K. (2011). Gold nanorod in reverse micelles: a fitting fusion to catapult lipase activity. *Chem. Commun.* 47:9864. 10.1039/c1cc12940f 21776523

[B148] Marcus R. A. Sutin N. (1985). Electron transfers in chemistry and biology. *Biochim. Biophys. Acta - Rev. Bioenerg.* 811 265–322. 10.1016/0304-4173(85)90014-X

[B149] Marquitan M. Mark M. D. Ernst A. Muhs A. Herlitze S. Ruff A. (2020). Glutamate detection at the cellular level by means of polymer/enzyme multilayer modified carbon nanoelectrodes. *J. Mater. Chem. B.* 8 3631–3639. 10.1039/C9TB02461A 31942595

[B150] Masini J. C. Svec F. (2017). Porous monoliths for on-line sample preparation: a review. *Anal. Chim. Acta* 964 24–44. 10.1016/j.aca.2017.02.002 28351637

[B151] Mateo C. Palomo J. M. Fernandez-Lorente G. Guisan J. M. Fernandez-Lafuente R. (2007). Improvement of enzyme activity, stability and selectivity via immobilization techniques. *Enzyme Microb. Technol.* 40 1451–1463. 10.1016/j.enzmictec.2007.01.018

[B152] Mateo C. Palomo J. M. van Langen L. M. van Rantwijk F. Sheldon R. A. (2004). A new, mild cross-linking methodology to prepare cross-linked enzyme aggregates. *Biotechnol. Bioeng.* 86 273–276. 10.1002/bit.20033 15083507

[B153] Matoori S. Leroux J.-C. (2020). Twenty-five years of polymersomes: lost in translation? *Mater. Horizons.* 7 1297–1309. 10.1039/C9MH01669D

[B154] Meller K. Pomastowski P. Szumski M. Buszewski B. (2017). Preparation of an improved hydrophilic monolith to make trypsin-immobilized microreactors. *J. Chromatogr. B Anal. Technol. Biomed. Life Sci.* 1043 128–137. 10.1016/j.jchromb.2016.08.032 27595484

[B155] Messina M. S. Messina K. M. M. Bhattacharya A. Montgomery H. R. Maynard H. D. (2020). Preparation of biomolecule-polymer conjugates by grafting-from using ATRP. RAFT, or ROMP. *Prog. Polym. Sci.* 100:101186 10.1016/j.progpolymsci.2019.101186 PMC7453843 32863465

[B156] Morgenstern J. Gil Alvaradejo G. Bluthardt N. Beloqui A. Delaittre G. Hubbuch J. (2018). Impact of polymer bioconjugation on protein stability and activity investigated with discrete conjugates: alternatives to PEGylation. *Biomacromolecules* 19 4250–4262. 10.1021/acs.biomac.8b01020 30222929

[B157] Morshed M. N. Behary N. Bouazizi N. Guan J. Chen G. Nierstrasz V. (2019). Surface modification of polyester fabric using plasma-dendrimer for robust immobilization of glucose oxidase enzyme. *Sci. Rep.* 9:15730. 10.1038/s41598-019-52087-8 31673063 PMC6823486

[B158] Moyano F. Falcone R. F. Mejuto J. C. Silber J. J. Correa N. M. (2010). Cationic reverse micelles create water with super hydrogen-bond-donor capacity for enzymatic catalysis: hydrolysis of 2-naphthyl acetate by α-Chymotrypsin. *Chem. A Eur. J.* 16 8887–8893. 10.1002/chem.201000437 20572177

[B159] Nadar S. S. Muley A. B. Ladole M. R. Joshi P. U. (2016). Macromolecular cross-linked enzyme aggregates (M-CLEAs) of α-amylase. *Int. J. Biol. Macromol.* 84 69–78. 10.1016/j.ijbiomac.2015.11.082 26675136

[B160] Naldi M. Èernigoj U. Štrancar A. Bartolini M. (2017). Towards automation in protein digestion: development of a monolithic trypsin immobilized reactor for highly efficient on-line digestion and analysis. *Talanta* 167 143–157. 10.1016/j.talanta.2017.02.016 28340705

[B161] Nelson J. M. Griffin E. G. (1916). Adsorption of invertase. *J. Am. Chem. Soc.* 38 1109–1115. 10.1021/ja02262a018

[B162] Ngo T. P. N. Zhang W. Wang W. Li Z. (2012). Reversible clustering of magnetic nanobiocatalysts for high-performance biocatalysis and easy catalyst recycling. *Chem. Commun.* 48 4585–4587. 10.1039/c2cc30953j 22450568

[B163] Nicoli R. Bartolini M. Rudaz S. Andrisano V. Veuthey J. L. (2008). Development of immobilized enzyme reactors based on human recombinant cytochrome P450 enzymes for phase I drug metabolism studies. *J. Chromatogr. A* 1206 2–10. 10.1016/j.chroma.2008.05.080 18556005

[B164] Nien P.-C. Tung T.-S. Ho K.-C. (2006). Amperometric glucose biosensor based on entrapment of glucose oxidase in a Poly(3,4-ethylenedioxythiophene) film. *Electroanalysis* 18 1408–1415. 10.1002/elan.200603552

[B165] Pachioni-Vasconcelos J. D. A. Lopes A. M. Apolinário A. C. Valenzuela-Oses J. K. Costa J. S. R. Nascimento L. D. O. (2016). Nanostructures for protein drug delivery. *Biomater. Sci.* 4 205–218. 10.1039/c5bm00360a 26580477

[B166] Palomo J. M. Muoz G. Fernández-Lorente G. Mateo C. Fernández-Lafuente R. Guisán J. M. (2002). Interfacial adsorption of lipases on very hydrophobic support (octadecyl-Sepabeads): Immobilization, hyperactivation and stabilization of the open form of lipases. *J. Mol. Catal. B Enzym.* 19 279–286. 10.1016/S1381-1177(02)00178-9

[B167] Parakhonskiy B. V. Yashchenok A. M. Konrad M. Skirtach A. G. (2014). Colloidal micro- and nano-particles as templates for polyelectrolyte multilayer capsules. *Adv. Coll. Interf. Sci.* 207 253–264. 10.1016/j.cis.2014.01.022 24594104

[B168] Parker E. K. Nielsen A. V. Beauchamp M. J. Almughamsi H. M. Nielsen J. B. Sonker M. (2019). 3D printed microfluidic devices with immunoaffinity monoliths for extraction of preterm birth biomarkers. *Anal. Bioanal. Chem.* 411 5405–5413. 10.1007/s00216-018-1440-9 30382326 PMC6494739

[B169] Peterson D. S. Rohr T. Svec F. Fréchet J. M. J. (2002). Enzymatic microreactor-on-a-chip: protein mapping using trypsin immobilized on porous polymer monoliths molded in channels of microfluidic devices. *Anal. Chem.* 74 4081–4088. 10.1021/ac020180q 12199578

[B170] Peterson D. S. Rohr T. Svec F. Fréchet J. M. J. (2003). Dual-function microanalytical device by in situ photolithographic grafting of porous polymer monolith: integrating solid-phase extraction and enzymatic digestion for peptide mass mapping. *Anal. Chem.* 75 5328–5335. 10.1021/ac034108j 14710809

[B171] Piro B. Dang L. A. Pham M. C. Fabiano S. Tran-Minh C. (2001). A glucose biosensor based on modified-enzyme incorporated within electropolymerised poly(3,4-ethylenedioxythiophene) (PEDT) films. *J. Electroanal. Chem.* 512 101–109. 10.1016/S0022-0728(01)00595-2

[B172] Pitzalis F. Carucci C. Naseri M. Fotouhi L. Magner E. Salis A. (2018). Lipase encapsulation onto ZIF-8: a comparison between biocatalysts obtained at low and high zinc/2-methylimidazole molar ratio in aqueous medium. *Chemcatchem* 10 1578–1585. 10.1002/cctc.201701984

[B173] Qian D. Nakamura C. Zorin N. Miyake J. (2002). Hydrogenase-poly(viologen) complex monolayers and electrochemical properties in langmuir-blodgett films. *Coll. Surf. A Physicochem. Eng. Asp.* 198–200 663–669. 10.1016/S0927-7757(01)00974-8

[B174] Rahman M. A. Kwon N. H. Won M. S. Choe E. S. Shim Y. B. (2005). Functionalized conducting polymer as an enzyme-immobilizing substrate: an amperometric glutamate microbiosensor for in vivo measurements. *Anal. Chem.* 77 4854–4860. 10.1021/ac050558v 16053298

[B175] Rehman S. Bhatti H. N. Bilal M. Asgher M. (2016). Cross-linked enzyme aggregates (CLEAs) of *Pencilluim notatum* lipase enzyme with improved activity, stability and reusability characteristics. *Int. J. Biol. Macromol.* 91 1161–1169. 10.1016/j.ijbiomac.2016.06.081 27365121

[B176] Ren L. Lv J. Wang H. Cheng Y. (2020). A coordinative dendrimer achieves excellent efficiency in cytosolic protein and peptide delivery. *Angew. Chem. Int. Edn.* 59 4711–4719. 10.1002/anie.201914970 31863674

[B177] Rodrigues R. C. Ortiz C. Berenguer-Murcia A. Torres R. Fernández-Lafuente R. (2013). Modifying enzyme activity and selectivity by immobilization. *Chem. Soc. Rev.* 42 6290–6307. 10.1039/c2cs35231a 23059445

[B178] Rodriguez-Abetxuko A. Morant-Minana M. C. Knez M. Beloqui A. (2019a). Carrierless immobilization route for highly robust metal-organic hybrid enzymes. *ACS Omega* 4 5172–5179. 10.1021/acsomega.8b03559

[B179] Rodriguez-Abetxuko A. Sánchez-deAlcázar D. Cortajarena A. L. Beloqui A. (2019b). A versatile approach for the assembly of highly tunable biocatalytic thin films. *Adv. Mater. Interf.* 6 1–9. 10.1002/admi.201900598

[B180] Rodriguez-Abetxuko A. Morant-Miñana M. C. López-Gallego F. Yate L. Seifert A. Knez M. (2018). Imidazole-grafted nanogels for the fabrication of organic-inorganic protein hybrids. *Adv. Funct. Mater.* 28:1803115 10.1002/adfm.201803115

[B181] Rodriguez-Abetxuko A. Muñumer P. Okuda M. Calvo J. Knez M. Beloqui A. (2020). Nanoconfined (Bio)catalysts as efficient glucose-responsive nanoreactors. *Adv. Funct. Mater.* 2002990 10.1002/adfm.202002990

[B182] Sahutoglu A. S. Akgul C. (2015). Immobilisation of *Aspergillus oryzae* α-amylase and *Aspergillus niger* glucoamylase enzymes as cross-linked enzyme aggregates. *Chem. Pap.* 69 433–439. 10.1515/chempap-2015-0031

[B183] Sakai S. Antoku K. Yamaguchi T. Kawakami K. (2008). Development of electrospun poly(vinyl alcohol) fibers immobilizing lipase highly activated by alkyl-silicate for flow-through reactors. *J. Memb. Sci.* 325 454–459. 10.1016/j.memsci.2008.08.008

[B184] Sakr O. S. Borchard G. (2013). Encapsulation of enzymes in layer-by-layer (LbL) structures: latest advances and applications. *Biomacromolecules* 14 2117–2135. 10.1021/bm400198p 23763594

[B185] Sánchez-deAlcázar D. Velasco-Lozano S. Zeballos N. López-Gallego F. Cortajarena A. L. (2019). Biocatalytic protein-based materials for integration into energy devices. *Chembiochem* 20 1977–1985. 10.1002/cbic.201900047 30939214

[B186] Sassolas A. Blum L. J. Leca-bouvier B. D. (2012). Immobilization strategies to develop enzymatic biosensors. *Biotechnol. Adv.* 30 489–511. 10.1016/j.biotechadv.2011.09.003 21951558

[B187] Scodeller P. Williams F. J. Calvo E. J. (2014). XPS analysis of enzyme and mediator at the surface of a layer-by-layer self-assembled wired enzyme electrode. *Anal. Chem.* 86 12180–12184. 10.1021/ac503147c 25420228

[B188] Serwa R. Majkut P. Horstmann B. Swiecicki J. M. Gerrits M. Krause E. (2010). Site-specific PEGylation of proteins by a staudinger-phosphite reaction. *Chem. Sci.* 1 596–602. 10.1039/c0sc00324g

[B189] Sharma K. P. Collins A. M. Perriman A. W. Mann S. (2013). Enzymatically active self-standing protein-polymer surfactant films prepared by hierarchical self-assembly. *Adv. Mater.* 25 2005–2010. 10.1002/adma.201204161 23381887

[B190] Shen H. Song J. Zhou Z. Li M. Zhang R. Su P. (2019). DNA-directed immobilized enzymes on recoverable magnetic nanoparticles shielded in nucleotide coordinated polymers. *Ind. Eng. Chem. Res.* 58 8585–8596. 10.1021/acs.iecr.9b01341

[B191] Sintra T. E. Ventura S. P. M. Coutinho J. A. P. (2014). Superactivity induced by micellar systems as the key for boosting the yield of enzymatic reactions. *J. Mol. Catal. B Enzym.* 107 140–151. 10.1016/j.molcatb.2014.06.001

[B192] Sproß J. Sinz A. (2010). A Capillary monolithic trypsin reactor for efficient protein digestion in online and offline coupling to ESI and MALDI mass spectrometry. *Anal. Chem.* 82 1434–1443. 10.1021/ac9025362 20099804

[B193] Sueyoshi D. Anraku Y. Komatsu T. Urano Y. Kataoka K. (2017). Enzyme-loaded polyion complex vesicles as in vivo nanoreactors working sustainably under the blood circulation: characterization and functional evaluation. *Biomacromolecules* 18 1189–1196. 10.1021/acs.biomac.6b01870 28233988

[B194] Šulek F. Fernández D. P. Knez Ž Habulin M. Sheldon R. A. (2011). Immobilization of horseradish peroxidase as crosslinked enzyme aggregates (CLEAs). *Process Biochem.* 46 765–769. 10.1016/j.procbio.2010.12.001

[B195] Sun C. Cheng Y. Pan Y. Yang J. Wang X. Xia F. (2020). Efficient polymerase chain reaction assisted by metal-organic frameworks. *Chem. Sci.* 11 797–802. 10.1039/c9sc03202a PMC8145698 34123055

[B196] Sun X. L. Yang L. C. Chaikof E. L. (2008). Chemoselective immobilization of biomolecules through aqueous Diels-Alder and PEG chemistry. *Tetrahedron Lett.* 49 2510–2513. 10.1016/j.tetlet.2008.02.111 19568325 PMC2703444

[B197] Sureka H. V. Obermeyer A. C. Flores R. J. Olsen B. D. (2019). Catalytic biosensors from complex coacervate core micelle (C3M) thin films. *ACS Appl. Mater. Interf.* 11 32354–32365. 10.1021/acsami.9b08478 31441305

[B198] Suthiwangcharoen N. Nagarajan R. (2014). Enhancing enzyme stability by construction of polymer-enzyme conjugate micelles for decontamination of organophosphate agents. *Biomacromolecules* 15 1142–1152. 10.1021/bm401531d 24564717

[B199] Tao L. Mantovani G. Lecolley F. Haddleton D. M. (2004). α-aldehyde terminally functional methacrylic polymers from living radical polymerization: application in protein conjugation “pegylation.”. *J. Am. Chem. Soc.* 126 13220–13221. 10.1021/ja0456454 15479065

[B200] Teepoo S. Dawan P. Barnthip N. (2017). Electrospun chitosan-gelatin biopolymer composite nanofibers for horseradish peroxidase immobilization in a hydrogen peroxide biosensor. *Biosensors* 7:47. 10.3390/bios7040047 29036932 PMC5746770

[B201] Torabizadeh H. Tavakoli M. Safari M. (2014). Immobilization of thermostable α-amylase from Bacillus licheniformis by cross-linked enzyme aggregates method using calcium and sodium ions as additives. *J. Mol. Catal. B Enzym.* 108 13–20. 10.1016/j.molcatb.2014.06.005

[B202] Turecek P. L. Bossard M. J. Schoetens F. Ivens I. A. (2016). PEGylation of biopharmaceuticals: a review of chemistry and nonclinical safety information of approved drugs. *J. Pharm. Sci.* 105 460–475. 10.1016/j.xphs.2015.11.015 26869412

[B203] Vahidi A. K. Wang Z. Wong W. S. Y. Li Z. (2016). Immobilization of: O -acetylserine sulfhydrylase as a highly active and recyclable nanobiocatalyst: efficient synthesis of β-pyrazol-1-yl-l-alanine. *Catal. Sci. Technol.* 6 6286–6293. 10.1039/c6cy00755d

[B204] Varlas S. Foster J. C. Georgiou P. G. Keogh R. Husband J. T. Williams D. S. (2019). Tuning the membrane permeability of polymersome nanoreactors developed by aqueous emulsion polymerization-induced self-assembly. *Nanoscale* 11 12643–12654. 10.1039/c9nr02507c 31237603

[B205] Vázquez E. Aguilar A. E. Moggio I. Arias E. Romero J. Barrientos H. (2007). Immobilization of the enzyme β-lactamase by self-assembly on thin films of a poly(phenyleneethynylene) sequenced with flexible segments containing sulfur atoms. *Mater. Sci. Eng. C* 27 787–793. 10.1016/j.msec.2006.08.022

[B206] Velasco-Lozano S. López-Gallego F. Mateos-Díaz J. C. Favela-Torres E. (2016). Cross-linked enzyme aggregates (CLEA) in enzyme improvement - a review. *Biocatalysis* 1 166–177. 10.1515/boca-2015-0012

[B207] Vinita, Nirala N. R. Tiwari M. Prakash R. (2018). A nanoporous palladium(II) bridged coordination polymer acting as a peroxidase mimic in a method for visual detection of glucose in tear and saliva. *Microchim. Acta* 185 1–10. 10.1007/s00604-018-2776-8 29610983

[B208] Vishwanath S. K. Watson C. R. Huang W. Bachas L. G. Bhattacharyya D. (1997). Kinetic studies of site-specifically and randomly immobilized alkaline phosphatase on functionalized membranes. *J. Chem. Technol. Biotechnol.* 68 294–302. 10.1002/(sici)1097-4660(199703)68:3<294::aid-jctb637>3.0.co;2-h

[B209] Wang L. Zeng Y. Shen A. Fu Y. Zeng L. Hu J. (2016). Facile and controllable synthesis of triplex Au@Ag-Pt@infinite coordination polymer core-shell nanoparticles for highly efficient immobilization of enzymes and enhanced electrochemical biosensing activity. *RSC Adv.* 6 86025–86033. 10.1039/c6ra15293g

[B210] Wang Q. Cui J. Li G. Zhang J. Li D. Huang F. (2014). Laccase immobilized on a pan/adsorbents composite nanofibrous membrane for catechol treatment by a biocatalysis/adsorption process. *Molecules* 19 3376–3388. 10.3390/molecules19033376 24651612 PMC6271767

[B211] Wang Q. Peng L. Du Y. Xu J. Cai Y. Feng Q. (2013). Fabrication of hydrophilic nanoporous PMMA/O-MMT composite microfibrous membrane and its use in enzyme immobilization. *J. Porous Mater.* 20 457–464. 10.1007/s10934-012-9615-9

[B212] Wang S. Su P. Ding F. Yang Y. (2013). Immobilization of cellulase on polyamidoamine dendrimer-grafted silica. *J. Mol. Catal. B Enzym.* 89 35–40. 10.1016/j.molcatb.2012.12.011

[B213] Wang X. Lu X. Wu L. Chen J. (2015). 3D metal-organic framework as highly efficient biosensing platform for ultrasensitive and rapid detection of bisphenol A. *Biosens. Bioelectron.* 65 295–301. 10.1016/j.bios.2014.10.010 25461172

[B214] Wang Y. Wu C. (2018). Site-specific conjugation of polymers to proteins. *Biomacromolecules* 19 1804–1825. 10.1021/acs.biomac.8b00248 29722971

[B215] Wang Z. Liu T. Asif M. Yu Y. Wang W. Wang H. (2018). Rimelike structure-inspired approach toward in situ-oriented self-assembly of hierarchical porous mof films as a sweat biosensor. *ACS Appl. Mater. Interfaces* 10 27936–27946. 10.1021/acsami.8b07868 30058799

[B216] Wang Z. G. Wang J. Q. Xu Z. K. (2006). Immobilization of lipase from *Candida rugosa* on electrospun polysulfone nanofibrous membranes by adsorption. *J. Mol. Catal. B Enzym.* 42 45–51. 10.1016/j.molcatb.2006.06.004

[B217] Watanabe F. Kubo T. Kaya K. Hosoya K. (2009). Novel separation medium spongy monolith for high throughput analyses. *J. Chromatogr. A* 1216 7402–7408. 10.1016/j.chroma.2009.06.054 19577755

[B218] Wei Z. Fan P. Jiao Y. Wang Y. Huang Y. Liu Z. (2020). Integrated microfluidic chip for on-line proteome analysis with combination of denaturing and rapid digestion of protein. *Anal. Chim. Acta* 1102 1–10. 10.1016/j.aca.2020.01.025 32043988

[B219] Weltz J. S. Kienle D. F. Schwartz D. K. Kaar J. L. (2019). Dramatic increase in catalytic performance of immobilized lipases by their stabilization on polymer brush supports. *ACS Catal.* 9 4992–5001. 10.1021/acscatal.9b01176

[B220] Wen L. Gao A. Cao Y. Svec F. Tan T. Lv Y. (2016a). Layer-by-layer assembly of metal-organic frameworks in macroporous polymer monolith and their use for enzyme immobilization. *Macromol. Rapid Commun.* 37 551–557. 10.1002/marc.201500705 26806691

[B221] Wen L. Tan X. Sun Q. Svec F. Lv Y. (2016b). “Smart” molecularly imprinted monoliths for the selective capture and easy release of proteins. *J. Sep. Sci.* 39 3267–3273. 10.1002/jssc.201600576 27352958

[B222] Wilson L. Illanes A. Abián O. Pessela B. C. C. Fernández-Lafuente R. Guisán J. M. (2004). Co-aggregation of penicillin G acylase and polyionic polymers: an easy methodology to prepare enzyme biocatalysts stable in organic media. *Biomacromolecules* 5 852–857. 10.1021/bm0343895 15132672

[B223] Wright T. A. Page R. C. Konkolewicz D. (2019). Polymer conjugation of proteins as a synthetic post-translational modification to impact their stability and activity. *Polym. Chem.* 10 434–454. 10.1039/c8py01399c 31249635 PMC6596429

[B224] Wu X. Yang C. Ge J. (2017). Green synthesis of enzyme/metal-organic framework composites with high stability in protein denaturing solvents. *Bioresour. Bioprocess.* 4:24. 10.1186/s40643-017-0154-8 28596935 PMC5438438

[B225] Wu X. Yue H. Zhang Y. Gao X. Li X. Wang L. (2019). Packaging and delivering enzymes by amorphous metal-organic frameworks. *Nat. Commun.* 10 1–8. 10.1038/s41467-019-13153-x 31727883 PMC6856190

[B226] Xiong M. Zhang M. Liu Q. Yang C. Xie Q. Ke G. (2020). Biomineralized nanoparticles enable an enzyme-assisted DNA signal amplification in living cells. *Chem. Commun.* 56 2901–2904. 10.1039/c9cc09503a 32037435

[B227] Yakup Arica M. Bayramoglu G. (2004). Reversible immobilization of tyrosinase onto polyethyleneimine-grafted and Cu(II) chelated poly(HEMA-co-GMA) reactive membranes. *J. Mol. Catal. B Enzym.* 27 255–265. 10.1016/j.molcatb.2003.12.006

[B228] Yamaguchi H. Miyazaki M. Asanomi Y. Maeda H. (2011). Poly-lysine supported cross-linked enzyme aggregates with efficient enzymatic activity and high operational stability. *Catal. Sci. Technol.* 1 1256–1261. 10.1039/c1cy00084e

[B229] Yan M. Du J. Gu Z. Liang M. Hu Y. Zhang W. (2010). A novel intracellular protein delivery platform based on single-protein nanocapsules. *Nat. Nanotechnol.* 5 48–53. 10.1038/nnano.2009.341 19935648

[B230] Yan M. Ge J. Liu Z. Ouyang P. (2006). Encapsulation of single enzyme in nanogel with enhanced biocatalytic activity and stability. *J. Am. Chem. Soc.* 128 11008–11009. 10.1021/ja064126t 16925402

[B231] Yang X. Tang Q. Jiang Y. Zhang M. Wang M. Mao L. (2019). Nanoscale ATP-responsive zeolitic imidazole framework-90 as a general platform for cytosolic protein delivery and genome editing. *J. Am. Chem. Soc.* 141 3782–3786. 10.1021/jacs.8b11996 30722666

[B232] You Y. Xu D. Pan X. Ma X. (2019). Self-propelled enzymatic nanomotors for enhancing synergetic photodynamic and starvation therapy by self-accelerated cascade reactions. *Appl. Mater. Today* 16 508–517. 10.1016/j.apmt.2019.07.008

[B233] Yu A. Wang Y. Barlow E. Caruso F. (2005). *Mesoporous silica* particles as templates for preparing enzyme-loaded biocompatible microcapsules. *Adv. Mater.* 17 1737–1741. 10.1002/adma.200402045

[B234] Yuan J. Gaponik N. Eychmüller A. (2012). Application of polymer quantum dot-enzyme hybrids in the biosensor development and test paper fabrication. *Anal. Chem.* 84 5047–5052. 10.1021/ac300714j 22545809

[B235] Zdarta J. Jankowska K. Bachosz K. Kijeńska-Gawrońska E. Zgoła-Grześkowiak A. Kaczorek E. (2019a). A promising laccase immobilization using electrospun materials for biocatalytic degradation of tetracycline: effect of process conditions and catalytic pathways. *Catal. Today.* 348 127–136. 10.1016/j.cattod.2019.08.042

[B236] Zdarta J. Jankowska K. Wyszowska M. Kijeńska-Gawrońska E. Zgoła-Grześkowiak A. Pinelo M. (2019b). Robust biodegradation of naproxen and diclofenac by laccase immobilized using electrospun nanofibers with enhanced stability and reusability. *Mater. Sci. Eng. C* 103:109789. 10.1016/j.msec.2019.109789 31349507

[B237] Zdarta J. Meyer A. Jesionowski T. Pinelo M. Zdarta J. Meyer A. S. (2018). A general overview of support materials for enzyme immobilization: characteristics, properties, practical utility. *Catalysts* 8:92 10.3390/catal8020092

[B238] Zhang A. Meng X. Bao C. Zhang Q. (2020). In situ synthesis of protein-loaded hydrogels via biocatalytic ATRP. *Polym. Chem.* 11 1525–1532. 10.1039/c9py01815h

[B239] Zhang C. Dong X. Guo Z. Sun Y. (2018). Remarkably enhanced activity and substrate affinity of lipase covalently bonded on zwitterionic polymer-grafted silica nanoparticles. *J. Coll. Interf. Sci.* 519 145–153. 10.1016/j.jcis.2018.02.039 29494877

[B240] Zhang Y. Park K. Y. Suazo K. F. Distefano M. D. (2018). Recent progress in enzymatic protein labelling techniques and their applications. *Chem. Soc. Rev.* 47 9106–9136. 10.1039/c8cs00537k 30259933 PMC6289631

[B241] Zhang C. Wang X. Hou M. Li X. Wu X. Ge J. (2017). Immobilization on metal-organic framework engenders high sensitivity for enzymatic electrochemical detection. *ACS Appl. Mater. Interf.* 9 13831–13836. 10.1021/acsami.7b02803 28398720

[B242] Zhang J. He M. Nie C. He M. Pan Q. Liu C. (2019a). Biomineralized metal-organic framework nanoparticles enable enzymatic rolling circle amplification in living cells for ultrasensitive microRNA imaging. *Anal. Chem.* 91 9049–9057. 10.1021/acs.analchem.9b01343 31274280

[B243] Zhang J. Huang X. Zhang L. Si Y. Guo S. Su H. (2019b). Layer-by-layer assembly for immobilizing enzymes in enzymatic biofuel cells. *Sustain. Energy Fuels* 4 68–79. 10.1039/c9se00643e

[B244] Zhang Y. Wang Y. Tang Y. Li R. Ji Y. (2019). An online immobilized pepsin microreactor based on polymer monoliths for screening inhibitors from natural products. *Anal. Methods* 11 2465–2472. 10.1039/c9ay00343f

[B245] Zhang P. Sun F. Tsao C. Liu S. Jain P. Sinclair A. (2015). Zwitterionic gel encapsulation promotes protein stability, enhances pharmacokinetics, and reduces immunogenicity. *Proc. Natl. Acad. Sci. U.S.A.* 112 12046–12051. 10.1073/pnas.1512465112 26371311 PMC4593115

[B246] Zhang Y. Ge J. Liu Z. (2015). Enhanced activity of immobilized or chemically modified enzymes. *ACS Catal.* 5 4503–4513. 10.1021/acscatal.5b00996

[B247] Zhang P. Wang Q. Zhang J. Li G. Wei Q. (2014). Preparation of amidoxime-modified polyacrylonitrile nanofibers immobilized with laccase for dye degradation. *Fibers Polym.* 15 30–34. 10.1007/s12221-014-0030-5

[B248] Zhao S. Zou Y. Liu X. Zhang H. (2019). Ecofriendly construction of enzyme reactor based on three-dimensional porous cryogel composites. *Chem. Eng. J.* 361 286–293. 10.1016/j.cej.2018.12.101

[B249] Zhao X. Fan P.-R. Mo C.-E. Huang Y.-P. Liu Z.-S. (2020). Green synthesis of monolithic enzyme microreactor based on thiol-ene click reaction for enzymatic hydrolysis of protein. *J. Chromatogr. A* 1611:460618. 10.1016/j.chroma.2019.460618 31672267

[B250] Zheng G. Liu S. Zha J. Zhang P. Xu X. Chen Y. (2019). Protecting enzymatic activity via zwitterionic nanocapsulation for the removal of phenol compound from wastewater. *Langmuir* 35 1858–1863. 10.1021/acs.langmuir.8b02001 30080053

[B251] Zhong X. Qian Y. Huang J. Yang D. Deng Y. Qiu X. (2016). Fabrication of lignosulfonate vesicular reverse micelles to immobilize horseradish peroxidase. *Ind. Eng. Chem. Res.* 55 2731–2737. 10.1021/acs.iecr.5b04939

[B252] Zhou Z. Ju X. Zhou M. Xu X. Fu J. Li L. (2019). An enhanced ionic liquid-tolerant immobilized cellulase system via hydrogel microsphere for improving in situ saccharification of biomass. *Bioresour. Technol.* 294:122146. 10.1016/j.biortech.2019.122146 31536857

[B253] Zhu L. L. Zhu C. T. Xiong M. Jin C. Q. Sheng S. Wu F. A. (2019). Enzyme immobilization on photopatterned temperature-response poly (N-isopropylacrylamide) for microfluidic biocatalysis. *J. Chem. Technol. Biotechnol.* 94 1670–1678. 10.1002/jctb.5946

